# A *Plasmodium vivax* experimental human infection model for evaluating efficacy of interventions

**DOI:** 10.1172/JCI134923

**Published:** 2020-04-27

**Authors:** Katharine A. Collins, Claire Y.T. Wang, Matthew Adams, Hayley Mitchell, Greg J. Robinson, Melanie Rampton, Suzanne Elliott, Anand Odedra, David Khoury, Emma Ballard, Todd B. Shelper, Leonardo Lucantoni, Vicky M. Avery, Stephan Chalon, Joerg J. Moehrle, James S. McCarthy

**Affiliations:** 1 QIMR Berghofer Medical Research Institute, Brisbane, Queensland, Australia.; 2 Department of Medical Microbiology, Radboud University Medical Centre, Nijmegen, Netherlands.; 3 Queensland Paediatric Infectious Diseases Laboratory, Centre for Children’s Health Research, Brisbane, Queensland, Australia.; 4 Q-Pharm Pty Ltd, Brisbane, Queensland, Australia.; 5 Kirby Institute, University of New South Wales, Sydney, New South Wales, Australia.; 6 Discovery Biology, Griffith University, Brisbane, Queensland, Australia.; 7 Medicines for Malaria Venture, Geneva, Switzerland.; 8 Faculty of Medicine, University of Queensland, Brisbane, Queensland, Australia.

**Keywords:** Infectious disease, Vaccines, Malaria

## Abstract

**BACKGROUND:**

Interventions that interrupt *Plasmodium vivax* transmission or eliminate dormant *P*. *vivax* liver-stage parasites will be essential for malaria elimination. Development of these interventions has been hindered by the lack of *P*. *vivax* in vitro culture and could be accelerated by a safe and reproducible clinical model in malaria-naive individuals.

**METHODS:**

Healthy, malaria-naive adults were enrolled in 2 studies to assess the safety, infectivity, and transmissibility of a new *P*. *vivax* isolate. Participants (Study 1, *n* = 2; Study 2, *n* = 24) were inoculated with *P*. *vivax*–infected red blood cells to initiate infection, and were treated with artemether-lumefantrine (Study 1) or chloroquine (Study 2). Primary endpoints were safety and infectivity of the new isolate. In Study 2, transmission to mosquitoes was also evaluated using mosquito feeding assays, and sporozoite viability was assessed using in vitro cultured hepatocytes.

**RESULTS:**

Parasitemia and gametocytemia developed in all participants and was cleared by antimalarial treatment. Adverse events were mostly mild or moderate and none were serious. Sixty-nine percent of participants (11/16) were infectious to *Anopheles* mosquitoes at peak gametocytemia. Mosquito infection rates reached 97% following membrane feeding with gametocyte-enriched blood, and sporozoites developed into liver-stage schizonts in culture.

**CONCLUSION:**

We have demonstrated the safe, reproducible, and efficient transmission of *P*. *vivax* gametocytes from humans to mosquitoes, and have established an experimental model that will accelerate the development of interventions targeting multiple stages of the *P*. *vivax* life cycle.

**TRIAL REGISTRATION:**

ACTRN12614000930684 and ACTRN12616000174482.

**FUNDING:**

(Australian) National Health and Medical Research Council Program Grant 1132975 (Study 1). Bill and Melinda Gates Foundation (OPP1111147) (Study 2).

## Introduction

*Plasmodium vivax* is the most globally widespread human malaria parasite, and the predominant cause of malaria outside of Africa (1). Although a major cause of morbidity, *P*. *vivax* infection has long been regarded as benign compared with *P*. *falciparum*. However, it has recently become widely recognized as a cause of severe, life-threatening, and fatal malaria infection (2, 3). As a consequence, there is renewed interest in developing *P*. *vivax*–specific control and elimination strategies (4). *P*. *vivax* is considered more difficult to control than *P*. *falciparum* due to the parasite’s unique biological features that increase its potential for transmission (5). Unlike *P*. *falciparum,* the transmissible stages of *P*. *vivax* (the gametocytes) appear early during blood-stage infection before the onset of symptoms, which increases the likelihood of transmission before treatment. *P*. *vivax* produces hypnozoites, which are dormant liver-stage parasites that cause relapses months to years after initial infection. Hypnozoites are reported to account for up to 80% of all *P*. *vivax* infections (6), thus providing repeated opportunities for onward transmission. In addition, *P*. *vivax* can be transmitted by a broad range of *Anopheles* vectors, many with exophilic and zoophilic tendencies, thus reducing the efficacy of conventional vector control measures (7). Therefore, as well as treating asexual parasites to control clinical illness, *P*. *vivax* control strategies must also target hypnozoites, and block transmission to have a marked impact on control and elimination (8).

The current recommended treatment for *P*. *vivax* is chloroquine or artemisinin-based combination therapy to clear asexual parasitemia, administered with the 8-aminoquinoline, primaquine, for 14 days to clear liver-stage hypnozoites (9). A single dose of tafenoquine recently demonstrated equivalent efficacy against hypnozoites with the potential to substantially improve treatment compliance. However, wide-scale deployment of these drugs to achieve meaningful public health impact is complicated by the need to screen for glucose-6-phosphate dehydrogenase deficiency, and safer alternatives are needed (10).

A *P*. *vivax* transmission–blocking vaccine (TBV) could interrupt transmission from primary infections, relapses, and also asymptomatic infections that remain undiagnosed and transmissible for a prolonged period. A TBV would reduce morbidity and mortality by preventing both new clinical infections and hypnozoite formation (11, 12). The inability to continuously culture *P*. *vivax* parasites in vitro and the difficulties in using animal models (8) have hampered development of interventions specifically targeting *P*. *vivax* hypnozoites and gametocytes. The production of gametocytes for evaluation of TBVs and sporozoites for liver-stage hypnozoite assays is limited to endemic settings where natural gametocyte carriers are available. Thus, a safe and reproducible in vivo model of human-to-mosquito *P*. *vivax* transmission in malaria-naive volunteers would accelerate development and early clinical evaluation of transmission-blocking interventions. Moreover, sporozoites generated from mosquitoes fed on gametocytes collected from unvaccinated volunteers during these studies could be used to evaluate interventions that target hypnozoites.

*P. vivax* experimental human infection studies, termed controlled human malaria infection (CHMI) or volunteer infection studies (VIS), have been established where malaria infections are initiated either by sporozoite inoculation or by the induced blood-stage malaria (IBSM) model (13). To date, none of these studies have demonstrated efficient *P*. *vivax* transmission from humans to mosquitoes. The IBSM model uses cryopreserved and characterized *P*. *vivax–*infected red blood cells (RBCs) to initiate infection. There have been only 2 previous *P*. *vivax* IBSM studies (both conducted at our center), in which a total of 8 adults were infected with a *P*. *vivax* isolate from the Solomon Islands; however, efficient transmission to mosquito was not achieved (14, 15). These studies were the first experimental infection of humans with blood-stage *P*. *vivax* using the modern IBSM model (deliberate infection with *P*. *vivax* was practiced between the 1920s and 1970s when malariotherapy was used for syphilis treatment [ref. 16], as well as in experimental studies with US prisoners [ref. 17]). Here, we evaluate the safety, tolerability, and infectivity of a new *P*. *vivax* isolate bank from India and describe a clinical model for evaluating the efficacy of blood-stage schizonticides and transmission-blocking interventions that can be exploited to facilitate the evaluation of *P*. *vivax* liver-stage interventions.

## Results

**Figure 1:**
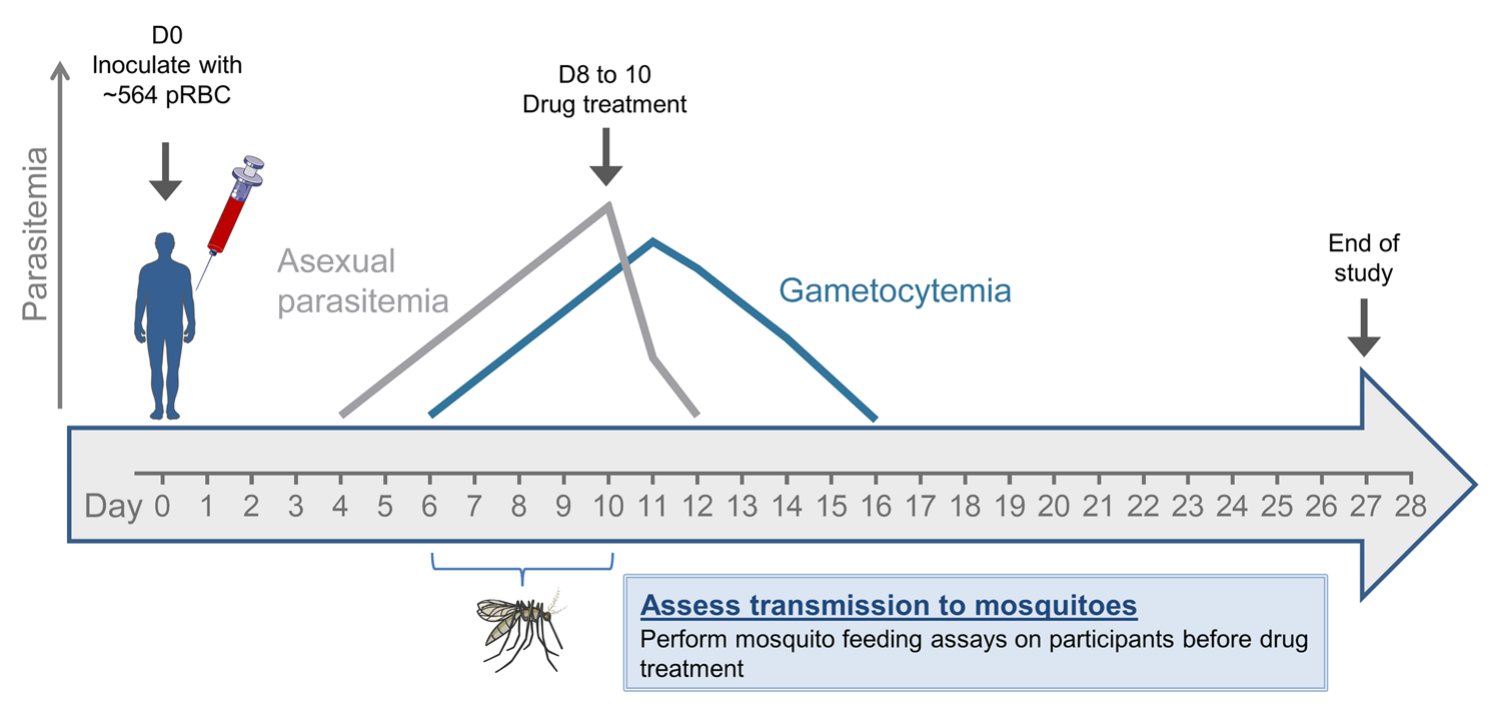
Study design schematic. Malaria-naive volunteers were inoculated with *P*. *vivax*–infected RBCs (pRBCs) on day 0 (D0). Asexual parasitemia and gametocytemia were evaluated from day 4 and continued until the end of study. Participants in Study 1 started artemether-lumefantrine treatment on day 8 (*n* = 2). Participants in Study 2 started chloroquine treatment on day 8 (*n* = 8), day 9 (*n* = 1), or 10 (*n* = 15). For Study 2, mosquito feeding assays were performed between day 6 and day 10 by direct feeding (allowing mosquitoes to feed on participants by live bite), or by membrane feeding on venous blood. D, day relative to inoculation (day 0); pRBC: *P*. *vivax* parasite–infected RBCs.

**Figure 2:**
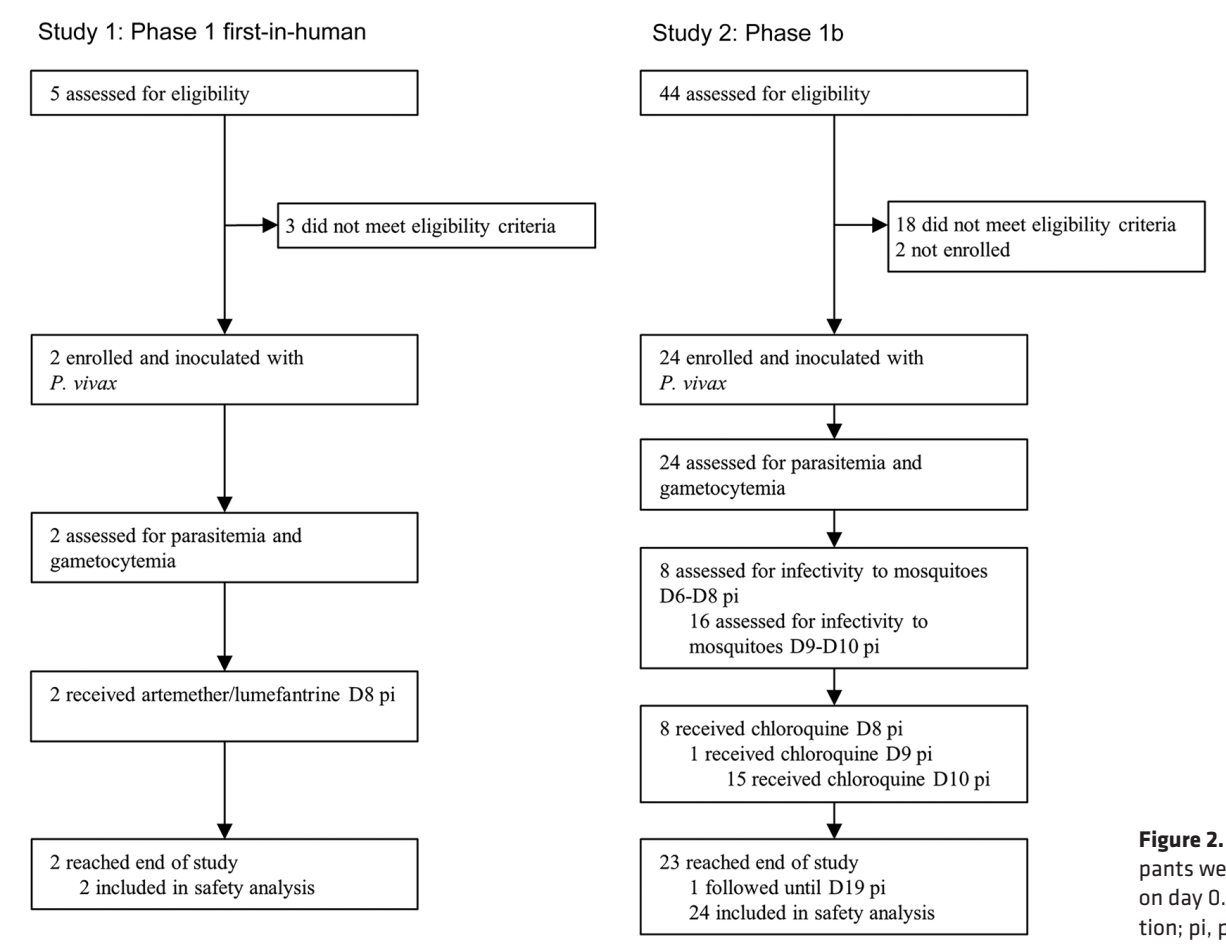
Study profile. All participants were inoculated with *P*. *vivax* on day 0. D, day relative to inoculation; pi, postinoculation.

Twenty-six malaria-naive volunteers were enrolled in 2 clinical trials: Study 1 (*n* = 2) undertaken from October 8, 2014, to January 8, 2015, and Study 2 (*n* = 24) undertaken from February 22, 2016, to May 21, 2017 (Figures 1 and 2). Baseline characteristics of participants are presented in Table 1.

All participants were inoculated with an estimated 564 viable *P*. *vivax* parasites, and the experimental infection was generally well tolerated. In Study 1, 14 adverse events (AEs) were reported: 12 attributed to malaria (headache, fever, myalgia, arthralgia, presyncope, rigors), 1 deemed possibly related to artemether-lumefantrine (somnolence), and 1 not related to malaria or artemether-lumefantrine (headache 49 days after treatment) (Table 2 and Supplemental Table 5). Most AEs resolved within 24 hours of treatment with paracetamol, except 2 intermittent headaches that resolved in 4 days and 8 days, and right knee pain that resolved in 4 days. All AEs were mild (*n* = 13/14; 92.9%) or moderate (*n* = 1/14; 7.1%) in severity. In Study 2, 355 AEs were reported (Table 2 and Supplemental Table 5). A total of 296 (83.4%) were related to malaria, and of these, 8 (2.3%) were concurrently deemed possibly related to chloroquine. Eleven (3.1%) AEs were related to direct skin feeding (DFA) (reaction at site of mosquito bite); the remaining AEs were attributed to other causes. Most AEs were mild (250/355; 70.4%) or moderate (98/355; 27.6%) in severity. Four severe AEs occurred and all were attributed to malaria: reduced neutrophil count (0.65 × 10^9^/L), chills, elevated alanine aminotransferase (peak 6.9 × ULN), and arthralgia. No serious AEs were reported in either trial.

**Figure 3:**
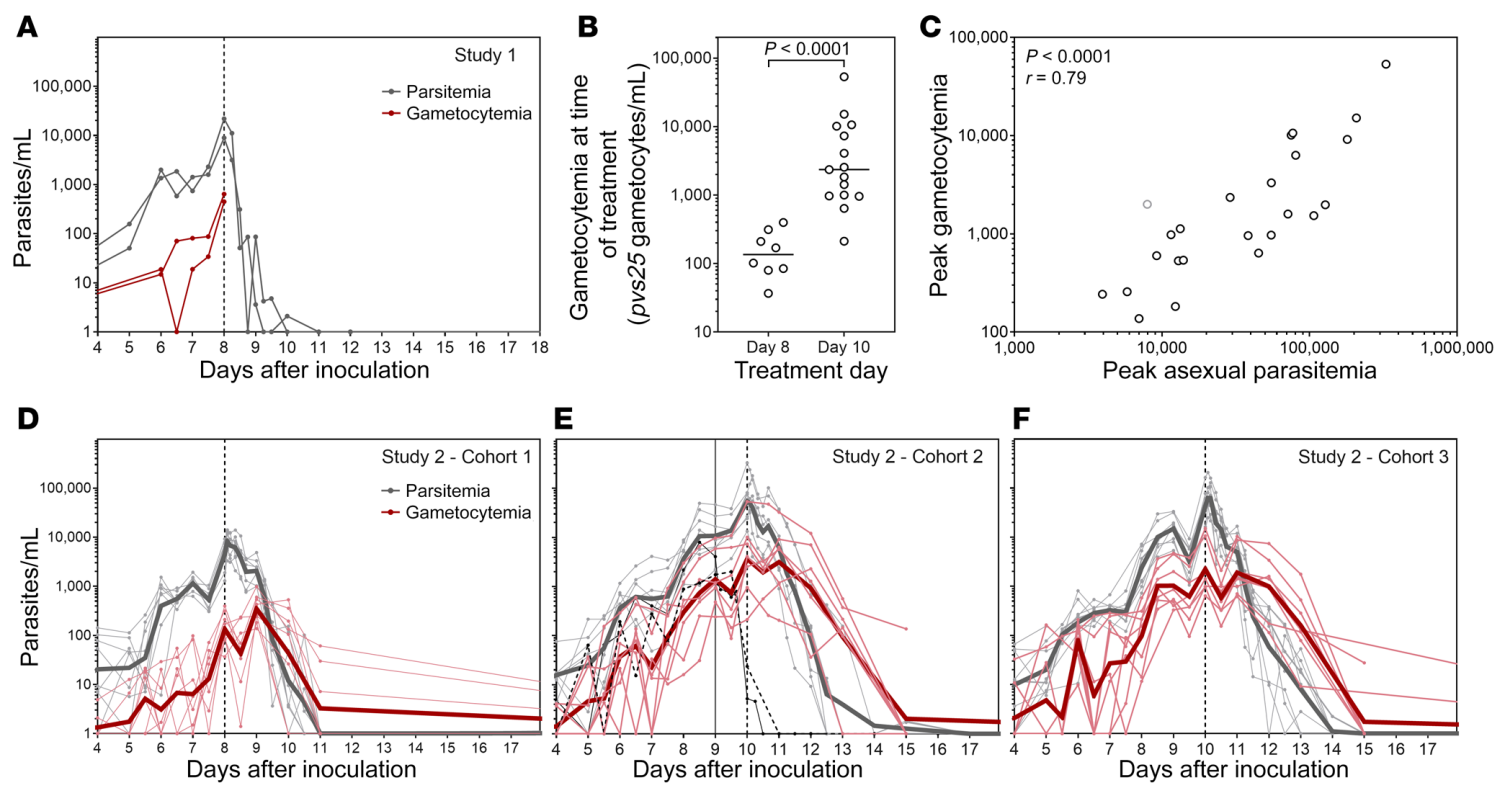
Parasitemia and gametocytemia. Participants (*n* = 26) were experimentally infected with *P*. *vivax* on day 0. Parasitemia was measured by 18S qPCR and gametocytemia measured by *pvs25* qRT-PCR for Study 1 (*n* = 2) (**A**), and Study 2 (*n* = 24) (**D**–**F**). Grey lines, parasitemia; red lines, gametocytemia. Thin lines show individual participant data and thick lines show the geometric mean. Initiation of treatment is indicated by the vertical lines. Treatment was initiated on day 8 for Study 1 (*n* = 2) and Study 2 cohort 1 (*n* = 8), or day 10 for Study 2 cohorts 2 and 3 (*n* = 15). Participant 205 (cohort 2; black lines) was treated on day 9 (vertical solid line). (**B**) Gametocytemia at time of treatment for Study 2 (*n* = 23) (compared by Mann-Whitney test). (**C**) Spearman’s correlation of peak asexual parasitemia and peak gametocytemia (*n* = 24). Participant 205 represented in gray.

All 26 participants developed blood-stage parasitemia. In Study 1, parasites were first detected by 18S quantitative PCR (18S qPCR) on day 5 in both participants. Parasitemia peaked at 21,836 parasites/mL and 8949 parasites/mL on the day of treatment (day 8), and was completely cleared following treatment with artemether-lumefantrine (Figure 3A). In Study 2, parasites were first detected by 18S qPCR in 21 of 24 participants on day 4, and in the remaining 3 participants on day 5. The course of parasite development did not differ between cohorts (Figure 3, D and F), and parasitemia was cleared in all participants in a median of 3 days after initiation of chloroquine treatment (range = 1.5–7.0 days).

Gametocytes were first detected (above 10 gametocytes/mL) on day 6 in Study 1 (Figure 3A) and between days 4 and 7 in Study 2, which was an average of 1.5 days (range = 0–3 days) after first detection of asexual parasites (Figure 3, D–F). Using the transcript number estimates per gametocyte published by Karl et al. (18) to convert *pvs25* transcripts/mL to gametocytes/mL, the peak gametocyte levels were 5.5% (median) of the peak asexual parasite levels, and gametocytemia correlated with asexual parasitemia (*P* < 0.0001) (Figure 3C). The course of gametocytemia followed the asexual parasitemia, but in Study 2 after chloroquine treatment, in contrast to immediate clearance of asexual parasites, clearance of gametocytes was delayed a further 24 hours.

In Study 2 cohort 1, median gametocytemia was 136 gametocytes/mL at the time of treatment/last mosquito feeding assay, meaning only 0.14 to 0.68 gametocytes would be imbibed in a 1- to 5-μL mosquito blood meal, making transmission extremely unlikely. As a consequence, following review of the safety data and approval from the Safety Monitoring Committee the recommendation was made to delay treatment until day 10 in cohorts 2 and 3. This resulted in significantly higher median gametocytemia at the time of treatment/last mosquito feeding assay (2351 gametocytes/mL; *P* < 0.0001) compared with participants in cohort 1 (Figure 3B).

**Figure 4:**
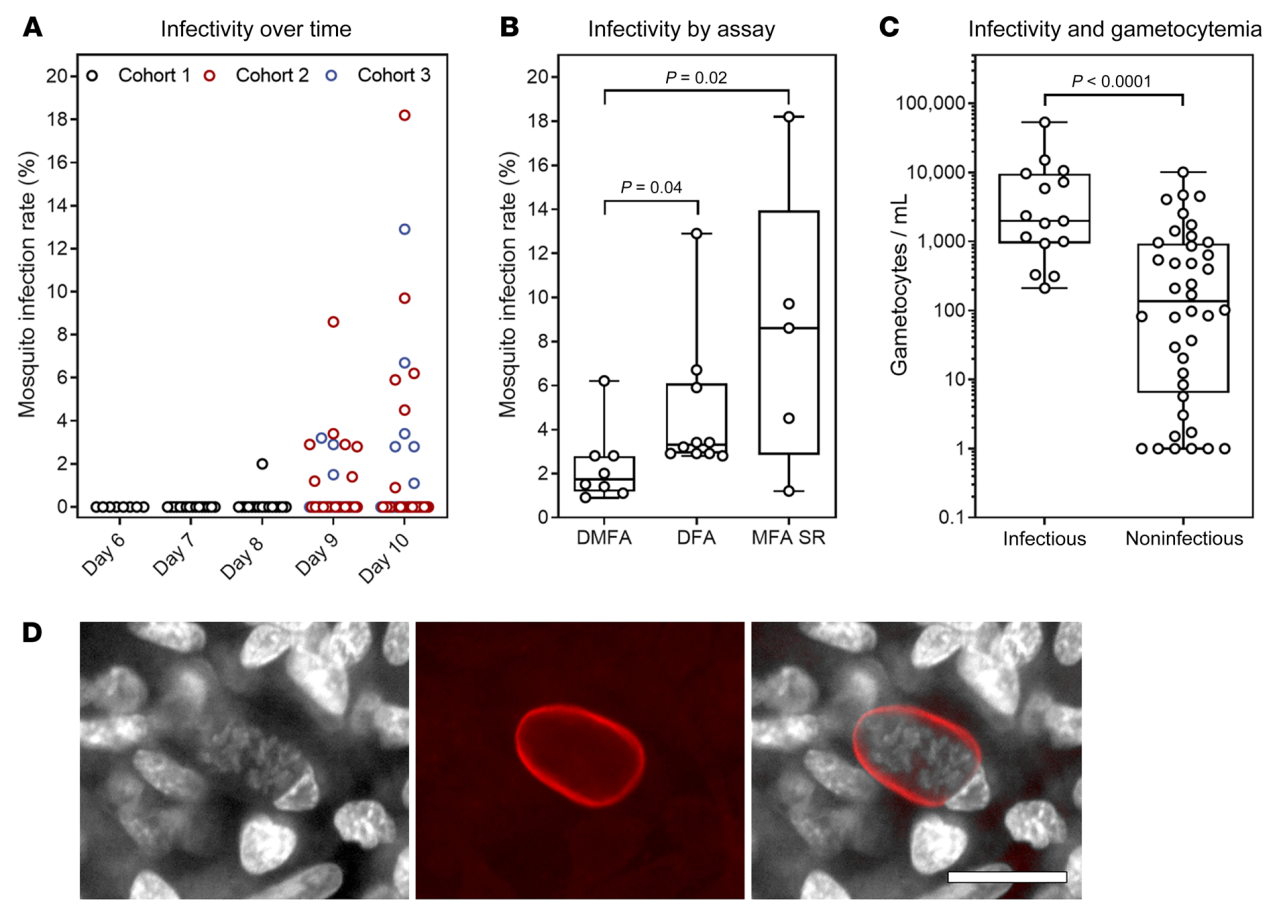
Infectivity to mosquitoes. Successful transmission was defined as at least 1 oocyst-positive mosquito as determined by 18S qPCR. Mosquito infection rate is reported as prevalence of infection (percentage of mosquitoes infected per feeding assay). (**A**) Prevalence of mosquito infection in all feeding assays in Study 2 at each time point (*n* = 113). (**B**) Prevalence of mosquito infection in successful feeding assays, by feeding assay type (*n* = 37). Groups compared by Kruskal-Wallis test with Dunn’s multiple comparison test. (**C**) The gametocytemia for participant samples that were infectious compared with samples that were noninfectious (*n* = 54). Groups compared by Mann-Whitney test. Box plots indicate the median and whiskers show the minimum and maximum. (**D**) Representative image from of a *P*. *vivax* liver-stage schizont stained with UIS4 and Hoechst33342 following incubation of sporozoites with HC-04 culture for 7 days (left, white channel, Hoechst33342; middle, red channel, Alexa Fluor 488–conjugated UIS4 antibody; right, merge). Image taken at ×40 magnification. Scale bar: 20 μm. Sporozoites were obtained by feeding mosquitoes on enriched gametocytes collected on day 10 from participants in cohort 3 (Supplemental Material).

The optimal times for mosquito feeding were days 9 and 10, when 69% (11/16) of participants were infectious to mosquitoes (Table 3 and Supplemental Table 6; supplemental material available online with this article; https://doi.org/10.1172/JCI134923DS1). Participants were not infectious on days 6 and 7 (0/8), and only one participant was infectious on day 8 (1/8). The rate of mosquito infection was highest on day 10 (Figure 4A; median on day 10 = 5.2%; IQR 2.8–8.9). Direct skin feeding resulted in higher mosquito infection rates (median = 3.3%; IQR 2.9–6.1) than direct membrane feeding with whole blood (median = 1.8%; IQR 1.2–2.8; *P* = 0.04), and membrane feeding with serum replacement (median = 8.6%; IQR 2.8–13.9) also resulted in significantly higher mosquito infection rates than membrane feeding with whole blood (*P* = 0.02) (Figure 4B and Supplemental Table 6). Successful mosquito transmission was associated with gametocyte density, with gametocytemia being significantly higher in the infectious samples (median = 1993 gametocytes/mL) compared with the noninfectious samples (median = 136 gametocytes/mL; *P* < 0.0001) (Figure 4C).

To increase mosquito infection rates in this model, we enriched gametocytes over a percoll gradient either approximately 10-fold or approximately 40-fold to increase the density of gametocytes offered to mosquitoes in the membrane feeding assays (19). Very high levels of mosquito infection ranging from 26% (day 9) to 92% (day 10) were achieved following approximately 10-fold enrichment (Table 4). When gametocytes were enriched approximately 40-fold, the mosquito infection rate was 97%, with a mean of 7 oocysts (range 1–16) per midgut. Salivary gland sporozoites were detected 15 to 17 days after the feeding assay, with an average of 7635 sporozoites per mosquito following approximately 40-fold enrichment (Table 4). To assess viability, these sporozoites were collected from the mosquitoes and incubated with HC-04 hepatocyte cells in culture. Following 7 days of incubation, liver-stage schizonts were observed by staining the cells with UIS4 monoclonal antibody (Figure 4D).

## Discussion

We have demonstrated, for what we believe is the first time, the safe, reproducible, and efficient transmission of gametocytes during experimental *P*. *vivax* malaria infection in humans, thereby establishing a new clinical model for evaluating *P*. *vivax* transmission–blocking interventions. Moreover, we have demonstrated the potential to exploit this model to produce viable clonal sporozoites capable of hepatocyte infection that could be used to evaluate interventions targeting *P*. *vivax* liver-stage parasites.

The new *P*. *vivax* HMP013 inoculum was safe and well-tolerated. The isolate was generated from a donor with blood group O (RhD positive), overcoming the need to match study volunteers’ blood group to that of the inoculum. The number and severity of AEs were in line with safety outcomes from published malaria IBSM trials, 2 of which used *P*. *vivax* (13). The severity of the single case of elevated alanine aminotransferase is similar to that reported in other *P*. *vivax* studies (15). A comprehensive analysis of clinically significant transaminase elevations in *P*. *vivax* IBSM studies will be reported separately.

Gametocytemia was detected in all participants and appeared in circulation early during blood-stage infection — only 1 to 2 days after the first appearance of asexual parasites — consistent with reports of a shorter gametocyte maturation time for *P*. *vivax* compared with *P*. *falciparum* (14, 15). The majority of participants (11/16; 68.8%) were infectious to laboratory-reared *An stephensi* mosquitoes on days 9 and 10 after infection. This represents the first report of efficient *P*. *vivax* gametocyte transmission during experimental malaria infection. Transmission from humans to mosquitoes was previously attempted during a sporozoite-induced *P*. *vivax* experimental malaria infection study, but was unsuccessful despite detection of the *pvs25* gametocyte marker (20, 21). In our previous *P*. *vivax* IBSM study (15), the peak gametocytemia was 43 gametocytes/mL compared with 47,393 gametocytes/mL in the present study (Supplemental Figure 2 and Supplemental Material). Difficulty was experienced during the previous study with verification of mosquito infection by microscopy. Review of the photomicrographs by a number of expert oocyst microscopists from different laboratories indicated a lack of consensus about which, if any, were true oocysts and which were artefact. This ambiguity about the identification of mosquito infection led us to develop and validate the qPCR assay used here for high-throughput, sensitive, and accurate evaluation of midgut infection (22). It was also followed by a study detailing the difficulty with oocyst identification by microscopy (23). Moreover, similar structures identified later in the same QIMR laboratory were confirmed PCR negative. Although we are unable to verify by PCR the result of the previous study with the Solomon Island isolate, we believe based on the lack of consensus about the identification of oocysts together with the very low gametocytemia during that study that it is likely that the reported mosquito infection rate was an overestimate. The study presented here thus demonstrates higher levels of gametocytemia, reliable transmission to mosquitoes, and increased assay validity. The mosquito infection rates we observed in this current study (1%–18%) are comparable to those reported from asymptomatic natural gametocyte carriers who had a mean gametocyte density of 1323 gametocytes/mL and an average mosquito infection rate of 4.2% (21). We also observed increasing mosquito infection rates with increasing gametocytemia, consistent with data from natural infections (21, 24). Transmission was low (on day 8) or did not occur (on days 6 and 7) before day 9, likely due to the low gametocyte densities at the time of feeding. Gametocytemia was so low (less than 397 gametocytes/mL) that the chance of gametocytes being taken up in a 1- to 5-μL blood meal was extremely unlikely. Membrane feeds performed with gametocytes that had been enriched over a percoll gradient resulted in very high levels of transmission, further demonstrating the observed relationship between gametocyte density and transmission success.

Our model provides a new platform to evaluate factors governing efficient transmission and, in accordance with previous *P*. *vivax* studies, mosquito infection rates were higher via the natural route of infection compared with feeding mosquitoes on whole blood via a membrane (25, 26). This is potentially due to conditions during membrane feeding being suboptimal for efficient transmission, or because gametocytes may localize to subdermal capillaries for more efficient uptake. Consistent with previous reports (19, 26), we observed higher mosquito infection rates from membrane feeding with serum replacement than from direct membrane feeding on whole blood. This suggests that a component of the venous blood sample not present in vivo during skin feeding, such as anticoagulant, may inhibit transmission (19, 26, 27).

Mosquito infection rates were very high after membrane feeding with enriched gametocytes. Midgut oocyst infections developed into salivary gland sporozoites, and these sporozoites were able to infect and develop in human hepatocytes in vitro. This demonstrates the potential application of this model to facilitate the study of *P*. *vivax* liver stages.

A limitation of this study is the small sample size; further studies are needed to determine the true variability in *P*. *vivax* infection characteristics among study participants. An additional limitation is that the IBSM model does not mimic natural infection as it bypasses the liver stage of infection. However, this offers a safety advantage because it eliminates the risk of hypnozoite formation during liver-stage infections and the potential for relapse. IBSM offers other logistical and safety advantages over *P*. *vivax* sporozoite–induced VIS including (a) the ability to readily carry out IBSM studies in nonendemic countries, (b) prior knowledge of *P*. *vivax* genotype and drug sensitivity, (c) ability to carry out multiple studies with the same strain and dose, and (d) simplified trial design and conduct because all participants develop blood-stage parasitemia simultaneously.

In conclusion, we have demonstrated the safe, reproducible, efficient transmission of *P*. *vivax* gametocytes from healthy nonimmune participants to mosquitoes during experimental human malaria infection. This experimental model can be used for early clinical evaluation of drug and vaccine candidates, and could provide a source of sporozoites for the evaluation of *P*. *vivax* liver stages. This model will further our understanding of the biology of all stages of *P*. *vivax* infection and provide critical information for malaria control and elimination agendas.

## Methods

### Study design and participants.

Two single-center open-label clinical trials were undertaken at Q-Pharm Pty Ltd in Queensland, Australia: a phase 1 first-in-human pilot safety and infectivity study (Study 1), and a phase 1b human-to-mosquito transmission study (Study 2). Healthy, malaria-naive males and nonpregnant, nonlactating females aged between 18 and 55 years were eligible to participate. Study 1 was conducted with 2 participants inoculated 24 hours apart. Study 2 was undertaken as 3 cohorts of 8 participants. Due to recruitment limitations, cohort 2 was performed as cohort 2a (*n* = 6) and cohort 2b (*n* = 2), conducted separately (Figures 1 and 2).

### Procedures.

The *P*. *vivax* HMP013 isolate was collected in 2014 from a traveler (blood group O, RhD positive) returning to Australia from India who presented with malaria-related symptoms. Informed consent was obtained (under a protocol approved by the QIMR Berghofer and Royal Brisbane Women’s Hospital human research ethics committees), and 200 mL of blood was collected. The patient tested negative for blood-borne pathogens using a Red Cross donation protocol and the RBCs were cryopreserved as previously described (14). The cryopreserved bank tested negative for adventitious agents and was subject to whole-genome sequencing (28).

Each inoculum was prepared by aseptically thawing and washing a vial of cryopreserved RBCs and diluting to 2 mL with injectable saline. The number of viable parasites per inoculum was retrospectively determined to be 564 parasites (95% CI: 342–930) by 18S qPCR (Supplemental Table 3 and Supplemental Material). All participants were inoculated intravenously on day 0 and monitored daily for AEs and malaria. From day 4, parasitemia was measured by 18S qPCR (Supplemental Material and ref. 14) twice-daily until participants were admitted to the clinic for treatment (Supplemental Tables 1 and 2). Gametocyte development was measured by qRT-PCR for *pvs25* mRNA (Supplemental Material) from day 4 (14). Curative antimalarial treatment was administered on day 8 (Study 1 and Study 2 cohort 1) or day 10 (Study 2 cohorts 2 and 3, except participant 205 who was treated on day 9). Participants in Study 1 received oral artemether-lumefantrine, and participants in Study 2 received oral chloroquine (Supplemental Table 4). All participants were confirmed parasite negative at the end of study (Figures 1 and 2).

For Study 2, infectivity of gametocytes was evaluated using mosquito feeding assays between days 6 and 8 (cohort 1) or on days 9 and 10 (cohorts 2 and 3). All feeding assays were performed before drug treatment was initiated. Gametocytes were fed to *Anopheles stephensi* mosquitoes via DFAs (2 per participant), DMFAs with whole venous blood in lithium heparin anticoagulant (2–3 per participant), or membrane feeding assays with serum replacement (MFA-SR) (19). Exploratory membrane feeding assays were performed to investigate mosquito infection rates when fed on gametocytes enriched from participants’ blood over a percoll gradient (Supplemental Material). We determined transmission to mosquitoes by measuring midgut oocyst infections using the 18S qPCR assay (14, 22). Microscopy was used to visually confirm oocysts in a small random selection of midguts before qPCR (Supplemental Figure 3, A and B). Salivary gland sporozoite infections were assessed using microscopy 15 to 17 days after mosquito feeding (Supplemental Figure 3C). Sporozoite viability was determined by adding salivary gland sporozoites to HC-04 cells in culture in liver-stage invasion assays (Supplemental Material).

### Outcomes.

Primary endpoints were the safety (both studies) and infectivity (Study 1) of the *P*. *vivax* isolate in healthy, malaria-naive adults. Safety endpoint measures were the frequency and severity of AEs, and results of clinical laboratory tests, physical examinations, vital sign assessments, and electrocardiographs. Infectivity endpoint measures were parasitemia and gametocytemia growth profiles determined by 18S qPCR and *pvs25* qRT-PCR. A secondary endpoint in Study 2 was transmissibility of *P*. *vivax* gametocytes from humans to mosquitoes. Successful transmission was defined as at least 1 oocyst-positive mosquito per feeding assay, measured by 18S qPCR. Additional primary and secondary objectives were to characterize the pharmacokinetic-pharmacodynamic relationship between chloroquine concentration and clearance of blood-stage parasites. These will be reported separately.

### Data sharing statement.

Data collected for the primary and secondary objectives for this study will be available with other supporting documents (e.g., protocol and informed consent) after publication upon request with a data transfer agreement. Direct inquiries to the corresponding author. All methodologies are presented in this manuscript or the Supplemental Methods. Where details are given in brief the method is already published in the accompanying reference.

### Statistics.

Both trials were designed to assess the in vivo safety of the *P*. *vivax* isolate in the IBSM model. The first-in-human pilot study (Study 1) required only 2 participants. Study 2 was designed to assess the parasite-clearing activity of chloroquine. Normative data on log parasite clearance rate was used in sample size estimation from 18 IBSM studies involving 102 individuals with mean decay rate of 0.063 log parasites per hour and SD of 0.019. It was determined that a sample size of 20 participants has 80% power to identify a difference of 20% in mean decay rate compared with a reference standard as significant at 5% 2-sided significance based on a 1-sample *t* test. Statistical analysis was performed using GraphPad Prism version 8.2.1 (infectivity endpoints), and R version 3.3.3 (inoculum size and calibration of 18S qPCR). The D’Agostino-Pearson normality test was used to determine if continuous data were normally distributed. When comparing 2 groups of nonparametric data, the Mann-Whitney test was used. More than 2 groups of nonparametric data were compared by Kruskal-Wallis test with Dunn’s multiple comparison test. *P* less than 0.05 was considered statistically significant.

### Study approval.

Both studies were approved by the QIMR Berghofer Human Research Ethics Committee. Study 2 was also approved by the Australian Defence Human Research Ethics Committee. All participants met the eligibility criteria (Supplemental Methods) and gave written informed consent before inclusion in the study. The trials were registered with the Australian New Zealand Clinical Trials Registry (ACTRN12614000930684 and ACTRN12616000174482).

## Author contributions

KAC, JSM, and JJM conceived the study and designed the experiment. JSM, SE, AO, and SC provided clinical oversight. KAC, CYTW, MA, HM, GJR, and MR performed molecular and entomology experiments. VMA, LL, TBS, and KAC were responsible for liver-stage assays. KAC, DK, and EB analyzed data. KAC wrote the manuscript. All authors reviewed the manuscript and approved the final version.

## Supplementary Material

Supplemental data

ICMJE disclosure forms

## Figures and Tables

**Table 3:**
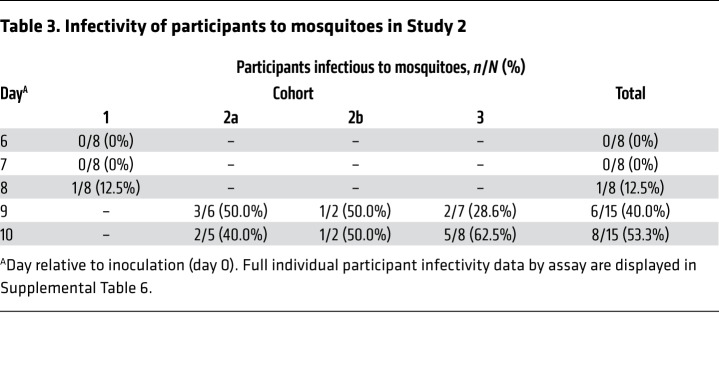
Infectivity of participants to mosquitoes in Study 2

**Table 2:**
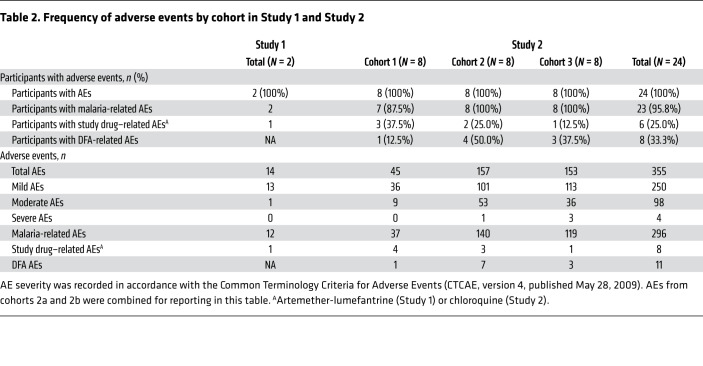
Frequency of adverse events by cohort in Study 1 and Study 2

**Table 1:**
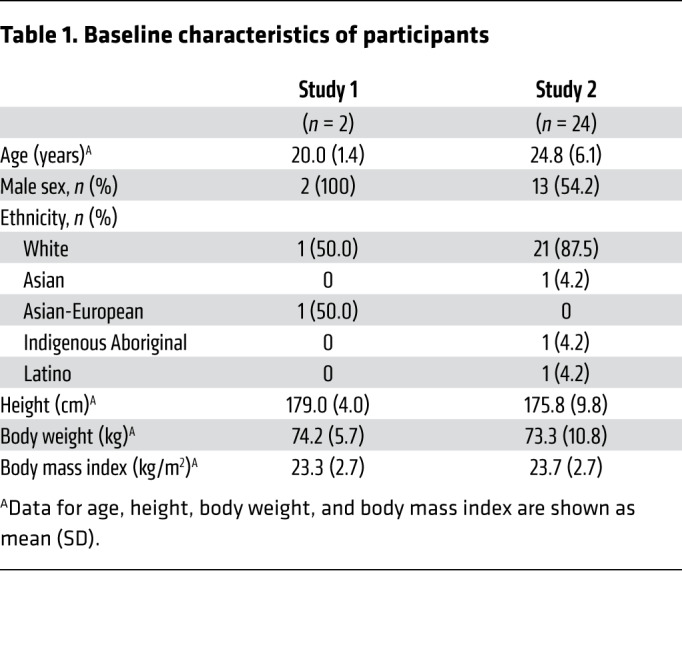
Baseline characteristics of participants

**Table 4:**
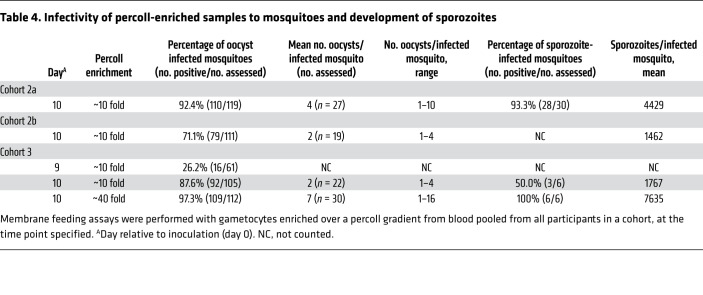
Infectivity of percoll-enriched samples to mosquitoes and development of sporozoites

## Version 1 02/11/2020

In-Press Preview

## Version 2 04/27/2020

Electronic publication

## Version 3 06/01/2020

Print issue publication
