# Supplementary material for: A Plasmodium vivax experimental human infection model for evaluating efficacy of interventions
Source: J Clin Invest. 2020 Apr 27;130(6):2920–7. doi: 10.1172/JCI134923 (PMC7259989; doi:10.1172/JCI134923)
Supplement: Supplemental data [file jci-130-134923-s359.pdf]

Supplementary information: A *Plasmodium vivax* experimental human infection model for evaluating interventions against multiple parasite stages

Table of contents

Eligibility criteria for study participants (Study 1) 3

Eligibility criteria for study participants (Study 2) 5

Table S1: Schedule of events (Study 1) 10

Table S2: Schedule of events (Study 2) 12

Molecular detection of blood stage parasites 13

Table S3: Primers and probes used in the qRT-PCR assays 14

Estimating inoculum size for volunteer infection studies using *P vivax* (Bank HMP013) (includes figure S1) 15

Anopheles stephensi mosquito colony and feeding assays 17

Liver invasion assays 18

Recalibration of the *P vivax* standard curve used in previous QIMR Berghofer *P vivax* IBSM studies (includes figure S2) 19

Table S4: Participant inoculation and chloroquine treatment (Study 2) 20

Table S5: All adverse events by system organ class and preferred term 22

Table S6: Participant infectivity to mosquitoes (Study 2) 23

Figure S3. Oocysts and sporozoites visualised by microscopy 24

References 25

SUPPLEMENTARY MATERIALS AND METHODS

Eligibility criteria for study participants (Study 1)

INCLUSION CRITERIA
Candidate participants who met all of the following criteria were eligible to be included in the study:

Demography
1. Adults (male or female) between 18 and 55 years of age inclusive, who did not live alone (from Day 0 until at least the end of the antimalarial drug treatment) and could be contactable and available for the duration of the trial (maximum of 4 months).
2. Body weight minimum 50·0 kg, body mass index between 18·0 and 32·0 kg/m^2, inclusive.

Health status
3. Certified as healthy by a comprehensive clinical assessment (detailed medical history and complete physical examination).
4. Normal vital signs after 10 min resting in supine position:
   - 95 mmHg < systolic blood pressure (SBP) <140 mmHg
   - 50 mmHg < diastolic blood pressure (DBP) <90 mmHg
   - 40 bpm < heart rate (HR) <100 bpm.
5. Normal standard 12-lead electrocardiogram (ECG) after 10 min resting in supine position; 120 ms<PR<210 ms, QRS<120 ms, QTcB<340 or QTcF≤450 ms with absence of second or third degree atrioventricular block or abnormal T wave morphology.
6. Laboratory parameters within the normal range, unless the Investigator considers an abnormality to be clinically irrelevant for healthy participants enrolled in this clinical investigation (for example, participants with asymptomatic mild hypercholesterolemia or glucose intolerance can be included). Serum creatinine, alkaline phosphatase, hepatic enzymes (aspartate aminotransferase, alanine aminotransferase), and total bilirubin (unless the participant has documented Gilbert syndrome) should not exceed the upper laboratory norm and haemoglobin must be higher than the lower limit of the normal range,
7. Female participants of childbearing potential, should be surgically sterile or using an insertable, injectable, transdermal, or combination oral contraceptive approved by the (US) FDA or (Australian) TGA combined with a barrier contraceptive through completion of the study and have negative results on a serum or urine pregnancy test done before administration of malaria inoculum.
8. Female participants of childbearing potential should be blood group Rh positive.

Regulations
9. Having given written informed consent prior to undertaking any study-related procedure.

EXCLUSION CRITERIA
Medical history and clinical status
1. Any history of malaria or participation in a previous malaria challenge study
2. Must not have travelled to or lived (>2 weeks) in a malaria-endemic country during the past 12 months or planned travel to a malaria-endemic country during the course of the study.
3. Has evidence of increased cardiovascular disease risk (defined as >10%, 5 year risk) as determined by the method of Gaziano et al.[1] Risk factors include sex, age, systolic blood pressure (mm/Hg), smoking status, body mass index (BMI, kg/m^2), and reported diabetes status.
4. History of splenectomy.
5. Pregnant or breastfeeding (all women will have a negative pregnancy test result prior to each study product administered).
6. Presence or history of drug hypersensitivity, or severe allergic reaction, anaphylaxis, or convulsions following any vaccination or infusion.
7. Presence of current or suspected serious chronic diseases such as cardiac or autoimmune disease (HIV or other immunodeficiencies), insulin-dependent and NIDDM diabetes, progressive neurological disease, severe malnutrition, acute or progressive hepatic disease, acute or progressive renal disease, psoriasis, rheumatoid arthritis, asthma, epilepsy or obsessive compulsive disorder, skin carcinoma excluding non-spreadable skin cancers such as basal cell and squamous cell carcinoma, schizophrenia, bipolar disorder, or other severe (disabling) chronic psychiatric diagnosis.

8. Participant with history of depression or receiving psychiatric drugs or who had been hospitalised within the past 5 years prior to enrolment for psychiatric illness, history of suicide attempt or confinement for danger to self or others.
9. Migraine, recurrent nausea, and/or vomiting (more than twice a month).
10. Known inherited genetic anomaly (known as cytogenetic disorders), e.g., Down syndrome.
11. Presence of acute infectious disease or fever (e.g., sublingual temperature ≥38·5°C) within the five days prior to inoculation with malaria parasites.
12. Evidence of acute illness within the four weeks before trial prior to screening.
13. Significant intercurrent disease of any type, in particular liver, renal, cardiac, pulmonary, neurologic, rheumatologic, or autoimmune disease by history, physical examination, and/or laboratory studies including urinalysis.
14. Participant has a clinically significant disease or any condition or disease that might affect drug absorption, distribution or excretion, e.g., gastrectomy, diarrhoea.
15. Participation in any investigational product study within the 12 weeks preceding the study.
16. Participation in any research study involving blood sampling, or blood donation to Red Cross (or other) blood bank during the 8 weeks preceding the reference drug dose in the study.
17. Participant unwilling to defer blood donations to the Australian Red Cross Blood Service (ARCBS) for 6 months.
18. Blood donation, any volume, within 1 month before inclusion.
19. Medical requirement for intravenous immunoglobulin or blood transfusions.
20. Participant who has ever received a blood transfusion, and females of child bearing potential who are blood group Rh negative.
21. Symptomatic postural hypotension, irrespective of the decrease in blood pressure, or asymptomatic postural hypotension defined as a decrease in systolic blood pressure ≥20 mmHg within 2–3 min when changing from supine to standing position.
22. History or presence of alcohol abuse (alcohol consumption more than 40 g per day) or drug habituation, or any prior intravenous usage of an illicit substance.
23. Smoking more than 5 cigarettes or equivalent per day and unable to stop smoking during the confinement period in the study.
24. Ingestion of any poppy seeds within the 24 h prior to the screening blood test (participants will be advised by phone not to consume any poppy seeds in this time period).
25. Excessive consumption of beverages containing xanthine bases, including Red Bull, chocolate, etc (e.g., more than 400 mg of caffeine per day (more than 4 cups or glasses per day).

Interfering substance

26. Any medication (including St John's Wort) within 14 days before inclusion or within 5 times the elimination half-life (whichever is longer) of the medication,
27. Any vaccination within the last 28 days.
28. Any recent or current therapy with an antibiotic or drug with potential antimalarial activity (tetracycline, azithromycin, clindamycin, hydroxychloroquine, etc.).
29. Unwillingness to abstain from consumption of grapefruit, or its juice, as well as quinine containing foods/beverages such as tonic water, lemon bitter, from inoculation (Day 0) to the end of the confinement period.

General conditions

30. Any participant who, in the judgment of the Investigator, is likely to be noncompliant during the study, or unable to cooperate because of a language problem or poor mental development.
31. Any participant in the exclusion period of a previous study according to applicable regulations.
32. Any participant who lives alone (from Day 0 until at least the end of the antimalarial drug treatment.
33. Any participant who cannot be contacted in case of emergency for the duration of the trial and up to 2 weeks following end of study visit.
34. Any participant who is the Investigator or any sub-investigator, research assistant, pharmacist, study coordinator, or other staff thereof, directly involved in conducting the study.
35. Any participant without a good peripheral venous access.

Biological status

36. Positive result on any of the following tests: hepatitis B surface (HBs Ag) antigen, anti-hepatitis B core antibodies (anti-HBc Ab), anti-hepatitis C virus (anti-HCV) antibodies, anti-human immunodeficiency virus 1 and 2 antibodies (anti-HIV1 and anti HIV2 Ab).

37. Any drug listed in Table 2 of the protocol in the urine drug screen unless there is an explanation acceptable to the medical investigator (e.g., the participant has stated in advance that they consumed a prescription or OTC product which contained the detected drug) and/or the participant has a negative urine drug screen on retest by the pathology laboratory.
38. Positive alcohol urine or breath test.

Specific to the study

39. Cardiac/QT risk:
    - A history of clinically significant ECG abnormalities.
    - Known pre-existing prolongation of the QTcB interval considered clinically significant,
    - Family history of sudden death or of congenital prolongation of the QTc interval or known congenital prolongation of the QTc interval or any clinical condition known to prolong the QTc interval.
    - History of symptomatic cardiac arrhythmias or with clinically relevant bradycardia.
    - Electrolyte disturbances, particularly hypokalaemia, hypocalcaemia or hypomagnesaemia,
    - Electrocardiogram (ECG) abnormalities in the standard 12-lead ECG (at screening) which in the opinion of the Investigator is clinically relevant or will interfere with the ECG analysis,
40. Known hypersensitivity to artemether-lumefantrine (Riamet®) or any of its excipients or 4-aminoquinolines, artemether or other artemisinin derivatives, lumefantrine, or other arylaminoalcohols.
41. Any history or presence of lactose intolerance.

On dosing days, and during the blood collection intervals:

1. Ingestion of any drug since the recruitment interview (other than the doses administered in this study) that, in the opinion of the Medical Investigator, could compromise the study.
2. Ingestion of any other drug, in the week prior to dosing or during the blood sampling period that, in the opinion of the Medical Investigator, could compromise the study, e.g., through pharmacokinetic or metabolic interactions, or analytical interference. However, the Medical Investigator may have permitted the use of paracetamol for the treatment of headache or other pain. If drug therapy other than paracetamol or drug specified in the protocol was required during the study periods, a decision to continue or discontinue the participant's participation was to be made by the Medical Investigator, based on the nature of the medication and the time the medication was taken.
3. Failure to conform to the requirements of the protocol.
4. Detection of any drug listed in the protocol in the urine drug screen unless there is an explanation acceptable to the medical investigator (e.g., the participant has stated in advance that they consumed a prescription or over-the-counter product, which contained the detected drug).
5. Vital signs outside the reference range and clinically significant.

Participants are requested to refrain from taking non-approved concomitant medication from recruitment until the conclusion of the study.

Participants who are excluded from participation on study days for any of the above reasons.

Eligibility criteria for study participants (Study 2)

INCLUSION CRITERIA

Candidate participants who met all of the following criteria were eligible to be included in Study 2:

Demography

1. Adults (male and non-pregnant, non-lactating female) between 18 and 55 years of age inclusive, who do not live alone (from Day 0 until at least the end of the antimalarial drug treatment) and will be contactable and available for the duration of the trial and follow up period (maximum of 6 weeks).
2. Body mass index between 18·0 and 32·0 kg/m^2, inclusive.

Health status

3. Certified as healthy by a comprehensive clinical assessment (detailed medical history and complete physical examination).
4. Normal vital signs after 5 min resting in supine position:
    - 90 mmHg ≤ systolic blood pressure (SBP) ≤ 140 mmHg
    - 50 mmHg ≤ diastolic blood pressure (DBP) ≤ 90 mmHg

- 40 bpm ≤ heart rate (HR) ≤ 100 bpm.

5. Normal standard 12-lead electrocardiogram (ECG) after 5 min resting in supine position, QTcF≤450 ms (males and females) with absence of second or third degree atrioventricular block or abnormal T wave morphology.
6. Laboratory parameters within the normal range, unless the Investigator considers an abnormality to be clinically irrelevant for healthy participants enrolled in this clinical investigation in accordance with approved clinically acceptable laboratory ranges documented prior to study start. More specifically for serum creatinine, hepatic transaminase enzymes (aspartate aminotransferase, alanine aminotransferase), and total bilirubin (unless the participant has documented Gilbert syndrome) should not exceed the approved acceptable ranges and haemoglobin must be equal or higher than the lower limit of the normal range.
7. As there is the risk of adverse effects of the study treatment drug (chloroquine), in pregnancy, it was important that any participant involved in this study did not get pregnant (refer to Section 6.7 of the protocol).
8. All participants were required to be Duffy blood group positive. Female participants of childbearing potential were required to be blood group Rh positive.
9. Female participants of childbearing potential (WOCBP) must also have adequate contraception in place for the duration of the study with adequate contraception defined as:
   - Stable hormonal contraception (with an approved oral, transdermal or depot regimen) for at least 3 months prior to screening i.e., oral contraceptives, either combined or progestogen alone, hormonal implantable contraception, vaginal ring, contraceptive patches.
   - Intrauterine (IUD) device or system in place for at least 3 months prior to screening.
   - Male partner sterilisation prior to the female participant's entry into the study, and this male is the sole partner for that participant.

Abstinent heterosexual female participants needed to agree to start a double method if they started a sexual relationship during the study.
Male participants needed to agree to use a double method of contraception including condom plus diaphragm or condom plus stable oral/transdermal/injectable hormonal contraceptive by female partner during the study.
Abstinent heterosexual male participants needed to agree to start a double method if they started a sexual relationship during the study.

Regulations

10. Having given written informed consent prior to undertaking any study-related procedure.

EXCLUSION CRITERIA

Participants who met any of the following criteria were not included in Study 2:

Medical history and clinical status

1. Any history of malaria or participation to a previous malaria challenge study
2. Any history of retinal abnormalities, disease of the retina or macula of the eye, visual field defects, hearing disorders (e.g., reduced hearing, tinnitus).
3. Must not have travelled to or lived (>2 weeks) in a malaria-endemic region during the past 12 months or planned travel to a malaria-endemic region during the course of the study (for endemic regions see https://map.ox.ac.uk/country-profiles/#!/).
4. Had evidence of increased cardiovascular disease risk (defined as >10%, 5 year risk for those greater than 35 years of age, as determined by the Australian Absolute Cardiovascular Disease Risk Calculator (http://www.cvdcheck.org.au/). Risk factors include sex, age, systolic blood pressure (mm/Hg), smoking status, total and HDL cholesterol (mmol/L), and reported diabetes status.
5. History of splenectomy.
6. Presence or history of drug hypersensitivity, or allergic disease diagnosed by an allergist/immunologist and/or treated by a physician for allergy or history of a severe allergic reaction, anaphylaxis or convulsions following any vaccination or infusion.
7. Presence of current or suspected serious chronic diseases such as cardiac or autoimmune disease (HIV or other immuno-deficiencies), insulin-dependent and non-insulin dependent diabetes, progressive neurological disease, severe malnutrition, acute or progressive hepatic disease, acute or progressive renal disease, porphyria, psoriasis, rheumatoid arthritis, asthma, epilepsy or obsessive compulsive disorder.
8. History of malignancy of any organ system (other than localised basal cell carcinoma of the skin or in situ cervical cancer), treated or untreated, within 5 years of screening, regardless of whether there is evidence of local recurrence or metastases.

9. Participants with history of schizophrenia, bipolar disorder, or other severe (disabling) chronic psychiatric diagnosis including depression or receiving psychiatric drugs or who has been hospitalised within the past 5 years prior to enrolment for psychiatric illness, history of suicide attempt or confinement for danger to self or others.
10. Frequent headaches and/or migraines, recurrent nausea, and/or vomiting (more than twice a month).
11. Presence of acute infectious disease or fever (e.g., sublingual temperature ≥38.5°C) within the 5 days prior to inoculation with malaria parasites.
12. Evidence of acute illness within the 4 weeks before trial prior to screening that the Investigator deemed may compromise participant safety.
13. Significant inter-current disease of any type, in particular liver, renal, cardiac, pulmonary, neurologic, rheumatologic, or autoimmune disease by history, physical examination, and/or laboratory studies including urinalysis.
14. Participant had a clinically significant disease or any condition or disease that might affect drug absorption, distribution or excretion: e.g., gastrectomy, diarrhoea.
15. Participation in any investigational product study within the 12 weeks preceding the study.
16. Blood donation, any volume, within 1 month before inclusion or participation in any research study involving blood sampling (more than 450 mL/unit of blood), or blood donation to Blood Service (ARCBS) or other during the 8 weeks preceding the treatment drug dose in the study.
17. Participant unwilling to defer blood donations to the Australian Red Cross Blood Service (ARCBS) for 6 months.
18. Medical requirement for intravenous immunoglobulin or blood transfusions.
19. Participant had ever received a blood transfusion.
20. Symptomatic postural hypotension at screening, irrespective of the decrease in blood pressure, or asymptomatic postural hypotension defined as a decrease in systolic blood pressure ≥20 mmHg within 2–3 min when changing from supine to standing position.
21. History or presence of alcohol abuse (alcohol consumption more than 40 g per day) or drug habituation, or any prior intravenous usage of an illicit substance.
22. Smoking more than 5 cigarettes or equivalent per day and unable to stop smoking for the duration of the study.
23. Ingestion of any poppy seeds within the 24 h prior to the screening blood test (participants were advised by phone not to consume any poppy seeds in this time period).
24. Excessive consumption of beverages containing xanthine bases, including Red Bull®, chocolate etc., more than 400 mg caffeine per day (equivalent to more than 4 cups per day).

Interfering substance

25. Any vaccination within the last 28 days.
26. Any corticosteroids, anti-inflammatory drugs, immunomodulators, or anticoagulants. Any participant who was currently receiving or had previously received immunosuppressive therapy, including systemic steroids including adrenocorticotrophic hormone (ACTH) or inhaled steroids in dosages which are associated with hypothalamic-pituitary-adrenal axis suppression such as 1 mg/kg/day of prednisone or its equivalent or chronic use of inhaled high potency corticosteroids (budesonide 800 µg per day or fluticasone 750 µg).
27. Any recent (<6 weeks) or current systemic therapy with an antibiotic or drug with potential anti-malarial activity (i.e., chloroquine, piperaquine, benzodiazepine, flunarizine, fluoxetine, tetracycline, azithromycin, clindamycin, etc.)

General conditions

28. Any participant who, in the judgement of the Investigator, was likely to be noncompliant during the study, or is unable to cooperate because of a language or mental deficit.
29. Any participant in the exclusion period of a previous study according to applicable regulations.
30. Any participant who lived alone (from Day 0 until at least the end of the antimalarial drug treatment).
31. Any participant who could not be contacted in case of emergency for the duration of the trial and up to 2 weeks following end of study visit.
32. Any participant who was the Investigator or any sub-investigator, research assistant, pharmacist, study coordinator, or other staff thereof, directly involved in conducting the study.
33. Any participant without a good peripheral venous access.

Biological status

34. Positive result on any of the following tests: hepatitis B surface (HBs Ag) antigen, anti-hepatitis B core antibodies (anti-HBc Ab), anti-hepatitis C virus (anti-HCV) antibodies, anti-human immunodeficiency virus 1 and 2 antibodies (anti-HIV1 and anti HIV2 Ab).

35. Any drug listed in Table 2 (Drug Screening) of the protocol in the urine drug screen unless there is an explanation acceptable to the medical investigator (e.g., the participant had stated in advance that they consumed a prescription or over-the-counter product, which contained the detected drug) and/or the participant has a negative urine drug screen on retest by the pathology laboratory. Any participant who tested positive for acetaminophen (paracetamol) at screening may still be eligible for study participation, at the Investigator's discretion.

Specific to the study

36. Cardiac/QT risk:
    - Family history of sudden death or of congenital prolongation of the QTc interval or known congenital prolongation of the QTc interval or any clinical condition known to prolong the QTc interval.
    - History of symptomatic cardiac arrhythmias or with clinically relevant bradycardia. Electrolyte disturbances, particularly hypokalaemia, hypocalcaemia, or hypomagnesaemia.
    - Electrocardiogram (ECG) abnormalities in the standard 12-lead ECG (at screening) that in the opinion of the Investigator is clinically relevant or will interfere with the ECG analyses on study.
37. Known hypersensitivity to chloroquine, or any of its excipients or 4-aminoquinolines, artemether or other artemisinin derivatives, lumefantrine, or other arylaminoalcohols.
38. Known severe reaction to mosquito bites other than local itching and redness.
39. Unwillingness to abstain from consumption of citrus (grapefruit, Seville orange, etc.) for ≥21 days prior to initiation of the study (inoculation, Day 0) and for the study duration.
40. Unwillingness to abstain from consumption of quinine containing foods/beverages such as tonic water, lemon bitter, from inoculation (Day 0) to the end of the antimalarial treatment.
41. Use of prescription drugs or non-prescription drugs and herbal supplements (such as St John's Wort), within 14 days or 5 half-lives (whichever was longer) prior to the inoculation administration. (Note: diazepam interferes with the analysis of blood levels of chloroquine and thus should not have been used for at least 8 weeks prior to administration of the study drug). If needed (i.e., an incidental and limited need) ibuprofen up to 1.2 g/day or paracetamol was acceptable up to 4 g/day (Cohort 1). Participants in Cohort 2 and 3 needed to agree to use either ibuprofen only up to 1.2 g/day or paracetamol only up to 4 g/day, depending on the cohort they were in or as advised by the investigator. Participants were required to notify the investigator before using a different treatment. Limited use of other non-prescription medications or dietary supplements not believed to affect participant safety or the overall results of the study may be permitted on a case-by-case basis following approval by the Sponsor in consultation with the Investigator.

Participants were requested to refrain from taking non-approved concomitant medication from recruitment until the conclusion of the study.

Participants who were excluded from participation on study days for any of the above reasons may have been eligible to participate on a postponed schedule if the Investigator considered this appropriate.

Procedures	Screen	IBSM challenge inoculum		Malaria monitoring	Artemether-lumefantrine treatment			Safety monitoring	Follow-up visit	Final visit or EOS
**Day**	**-D28 to -D2**	**D0**	**D1 to D6**	**From D7 until 18S qPCR positive, and then am and pm until admission**	**Admission ~D14**	**Confinement at clinical site**	**Exit from clinical site**	**After artemether-lumefantrine treatment**	**D28±3**	**D90±14**
Informed consent and eligibility	X	X								
Medical history	X	X							X	X
Physical examination [a]	X	X		X	X	X	X	X	X	X
Electrocardiogram [b]	X	X			X		X		X	X (if not done at D28)
Vital signs [c]	X	X		X	X	X	X	X	X	X
Hematology and biochemistry [d]	X	X			X		X	X	X	
Serology [e]	X								X	X
Red cell alloantibody	X								X	X
Urinalysis [f, h]	X	X							X	
Drug and alcohol screen [g]	X	X			X					
Phone call			X							
IBSM challenge		X								
Confinement at clinical site[i]					X	X				
**Artemether-lumefantrine treatment**						X[j]	X[j]	X[i]		
Adverse events		X	X	X	X	X	X	X	X	X
pvs25 qRT-PCR[j,k]		X		X	X	X	X	X	X	
Safety serum storage		X							X	X

D= Day relative to inoculation on Day 0, EOS=end of study visit, LFT=liver function test; PK=pharmacokinetic; qPCR=quantitative polymerase chain reaction; qRT-PCR=quantitative real-time PCR

[a] Physical examination was to be conducted as described in the protocol. Complete physical examination was to be performed at screening and then at D28 or end of study (EOS) if the participant was withdrawn before D28. An abbreviated physical examination was to be performed on the day of challenge, upon admission to the unit and at all morning and/or evening visits during confinement and during outpatient phases where symptoms of malaria were identified or if otherwise clinically indicated.

[b] Electrocardiogram (ECG): 12-lead electrocardiogram was to be recorded at screening (in triplicate) and on D0 pre malaria inoculum, on admission to the unit before artemether-lumefantrine dosing and then on exit from the unit, and at the D28 visit (or EOS if withdrawn before D28) or D90 if it was not done at D28.

[c] Vital signs: temperature (sublingual), respiratory rate, heart rate and blood pressure was to be measured at screening and on a daily basis at a minimum from D0 (excluding D1 and D6), D7 until confinement, three times per day during confinement and then as per the schedule until the final visit (D28/D90/EOS if participant was withdrawn before D90). Vital signs may have been measured on other visit days as indicated.
[d] Haematology and biochemistry tests were to be performed at Screening, D0 pre malaria inoculum, upon admission to the clinical unit prior to artemether-lumefantrine dosing, and upon exit of the unit, on the initial day of artemether-lumefantrine treatment and at follow up visits on D28/D90/EOS if the participant was withdrawn before D90. Reticulocytes and red cell antibodies were to be assessed at D0 baseline (reticulocytes) or screening visit (red cell antibodies) and D28 visit or EOS. Liver function was to be monitored on an outpatient basis 2 to 3 times per week following the completion of artemether-lumefantrine treatment and D28 visit as advised by the Principal Investigator.
[e] Viral and other serology: HIV, hepatitis B, hepatitis C, Epstein-Barr virus, cytomegalovirus, was to be assessed at screening, D28 and D90/EOS if withdrawn before D90.
[f] Urinalysis was to be conducted at screening, day of challenge, D28/EOS if participant was withdrawn before D28.
[g] Urine drug screen was to be performed at screening, D0, and upon entry to the clinical unit. Alcohol breath test was to be conducted at screening, D0 pre inoculation, and upon entry to the clinical unit.
[h] Pregnancy testing was to be conducted on all female participants: Serum test at screening and urine test on D0 pre malaria inoculum dosing, upon admission to the unit prior to artemether-lumefantrine dosing, and at follow up visits (D28 or EOS if participant was withdrawn before D28).
[i] Confinement period was to be at least 48 h (as advised by the clinical staff)
[j] Artemether-lumefantrine treatment from first clinical symptoms of malaria infection for the required period until completion of dosing
[k] qPCR: Baseline blood was to be collected per schedule for malaria parasite levels assessment. Blood was to be collected prior to inoculation on D0 and then daily from D7 until 18S qPCR positive for malaria, then twice-daily until artemether-lumefantrine dosing, at scheduled time points through confinement, and until 18S qPCR negative and D28/EOS if participant was withdrawn before D28. Bloods were to be collected am/pm, or am (i.e., 7:00 am–9:00 am and 7:00 pm–9:00 pm) as described in the protocol. Extra blood were to be collected for gametocyte specific PCR *pvs25*.

Table S1: Schedule of events (Study 1)

Events	Screening	Pre-inoculation screen	IBSM challenge inoculum	Monitoring for malaria using 18S rRNA qPCR		Mosquito feeding	Chloroquine treatment		Safety monitoring	Final visit or EOS
**Day**	**–D28 to –D3**	**–D3 to –D1**	**D0**	**Days 1, 2 & 3**	**D4 am until PCR +ve for malaria**	**~1-3 days before CQ dosing**	**Admission to clinical unit (D8 or D10)**	**72 h in clinical unit**	**Up to 21 days after CQ dosing**	**D28±3**
Informed consent	X		X							
Medical history, eligibility, and prior medications	X									
Physical examination [a]	X		X		X		X	X	X	X
ECG [b]	X		X				X	X		X
Vital signs assessment [c]	X		X		X		X	X	X	X
Hematology & biochemistry [d]	X	X					X	X	X	X
Serology [e]	X									X
Pregnancy test	X		X				X			X
Red cell alloantibody test	X									X
Urinalysis [h]	X	X								X
Urine drug screen [i]	X		X				X			
Alcohol breath test			X				X			
IBSM challenge inoculum	X		X							
Phone call or SMS	X			X						
Malaria clinical score assessment	X				X	X	X	X	X	
72 h confinement in clinical unit [j]	X						X	X		
Chloroquine treatment	X						X	X		
Adverse event monitoring	X		X	X	X	X	X	X	X	X
18S rRNA qPCR [l]	X		X		X	X	X	X	X	X
pvs25 qRT-PCR	X		X		X	X	X	X	X	X
Chloroquine PK blood sampling [m]	X						X	X	X	X
Membrane feeding assay [o]	X					X	X			
Direct feeding assay [o]	X					X	X			
Safety serum storage	X		X							X

CQ=chloroquine; D= Day relative to inoculation on Day 0, EOS=end of study visit, LFT=liver function test; PK=pharmacokinetic; qPCR=quantitative polymerase chain reaction; qRT-PCR=quantitative real-time PCR

[a] A complete physical examination (see Table 3 of the protocol) was to be performed at screening and the final visit (EOS). An abbreviated physical examination was to be performed D0 pre-inoculum, from D4 post-inoculum (if symptomatic), upon admission to the unit and at all morning and evening visits during confinement and where symptoms of malaria were identified.

[b] Single 12-lead electrocardiograms (ECGs) were recorded at screening, D0 pre-inoculum, on entry for confinement pre-chloroquine dose, 2, 4, 24, and 48 h after first chloroquine dose, and at D28±3 or EOS.
[c] Temperature (sublingual), respiratory rate, heart rate, and blood pressure were obtained at screening, pre- and post-inoculum, pre- and post-chloroquine doses, and prior to leaving the clinical unit. These vital signs were also measured at each outpatient visit and 3 times per day during confinement or when malaria symptoms were identified. Vital signs were also assessed at the final visit (Day 28±3 or EOS). Participants were required to remain semi-recumbent for 5 min prior to measurement of heart rate and blood pressure.
[d] Haematology and biochemistry samples were collected at screening, pre-inoculation evaluation (if required), pre-dose with chloroquine, following treatment at 72 h (exit from confinement), post-confinement at 120 h (LFT only), 168, 240, and 336 h, and at EOS (D28 ±3). See Table 2 in the protocol for specific haematology and biochemistry tests.
[e] Viral and other serology tests conducted at screening and EOS were HIV, hepatitis B, and hepatitis C. Tests for EBV, CMV, and toxoplasma were conducted only at investigator discretion.
[f] Urinalysis at screening and final visit (D28±3 or EOS).
[g] Urine drug screen at screening, D0 pre-inoculum, and at admission to the clinical unit pre-chloroquine dosing. For participants in cohorts 2a, 2b, and 3, an alcohol breath test was conducted at screening, D0 pre-inoculum, and at admission to the clinical unit pre-chloroquine dose.
[h] 72h confinement in clinical unit from D8 am (Cohort 1) or D10 am (cohorts 2a, 2b, and 3).Chloroquine treatment: individual participant treatment within 24 h if parasitaemia was equal to or greater than 20,000 parasites/mL (as measured by 18S rRNA qPCR) and malaria clinical score was greater than 6 or cohort treatment if 18S rRNA qPCR parasitaemia in all participants is equal to or greater than 20,000 parasites/mL.
[j] Blood samples were collected for18S rRNA qPCR on D0, daily from D4 (am), and twice-daily (am and pm) once when malaria-positive, until dosing with chloroquine. During confinement, blood was collected pre-chloroquine dose, post-chloroquine dose at 2, 4, 8, 12, 16, 24, 30, 36, 48, 60, and 72 h (exit from clinical unit).
[k] Chloroquine (and metabolite DECQ) PK blood sampling at pre-chloroquine, and then at 1, 2, 3, 4, 6, 12, 24, 48, 72, 96, 168, 240, and 480 h post-chloroquine administration.
[l] Thick blood films were optional and were not conducted in this study.
[m] Direct feeding assays and membrane feeding assays were conducted on D6 to 8.

Table S2: Schedule of events (Study 2)

Molecular detection of blood stage parasites

Nucleic acid extraction. Packed red blood cells (250 µL) mixed with PBS (250 µL) were stored in 400 µL AL buffer (Qiagen, Australia) and DNA was extracted using QIAmp DNA blood mini kit (Qiagen Australia) following the protocol previously described.[5] A second 250 µL aliquot of packed red blood cells was stored (1:5) in RNAprotect Cell Reagent (Qiagen, Australia) at -80°C until RNA extraction. RNA extraction was performed using RNeasy Plus Mini Kit (Qiagen, Australia) following manufacturer's instructions with treatments of DNase on-column digestion using RNase-Free DNase set (Qiagen, Australia) to eliminate genomic DNA.

Total parasite quantification. *P vivax* parasites were quantified using previously described *Plasmodium* genus-specific qPCR assay targeting DNA from the consensus 18S ribosomal RNA gene (rRNA).[6] The consensus qPCR assay is able to amplify five different *Plasmodium* species including *P falciparum, P vivax*, *P ovale, P malariae* and *P knowlesi*. In brief, each 25 µL PCR reaction mix contained 12·5 µL of Quantitect Probe PCR mix (Qiagen, Australia), 0·4 µM of each forward and reverse primer, 0·16 µM of Taqman probe and 5 µL of DNA template. Amplification was performed in a Rotorgene 3000 or Q instrument (Qiagen, Australia) under the following cycling conditions: 95°C activation for 15 min, 45 cycles of 95°C for 15 sec and 60°C for 60 sec.

Since continuous culture of *P vivax* parasites was not available, cultured *P falciparum* 3D7 parasites were quantified as previously described [2] and used to generate a standard curve. Five serial dilutions were prepared from the original quantified sample in uninfected human whole blood, with parasite concentrations ranging from $3{\cdot}19\times10^5$ to $3{\cdot}19\times10^1$ parasites/mL. The dilutions were extracted as previously described.[5] Six replicates of each dilution extract were analysed on 18S rDNA qPCR assay to construct a standard curve and a linear regression model was generated within Rotorgene Software (Qiagen, Australia). The regression model was imported to subsequent qPCR runs with calibrators to calculate parasite concentration, reported at parasites/mL of whole blood. To ensure parasite quantification using *P falciparum* as the standards reflects accurate estimates for *P vivax* and to account for slight difference of the qPCR assay binding between *P falciparum* and *P vivax* genomes, the parasitaemia was recalibrated using a *P vivax* blood sample. The level of parasitaemia in this sample had been precisely quantified by microscopy and was estimated to have 7,446 parasites/µL with 91% ring stage parasites. This sample was diluted 1 in 2 and a 10-fold dilution series made from the nucleic acid extract ranging from $3{\cdot}72\times10^6$ to 3·72 parasites/mL. Each sample was run in triplicate over three days (a total of 63 qPCR reactions). The 18S rDNA genome equivalents were determined in these samples using the qPCR assay. The study design was a randomised block design with the dilution series as treatments, daily runs as blocks, and technical replicates used to estimate intra-assay variability. An analysis of variance on the residual difference in $\log_{10}$ (estimated parasites/mL) and $\log_{10}$ (actual *P vivax* parasites/mL) was used to obtain mean and 95% CIs for the scaling factor to correct for *P vivax*. The scaling factor applied to all samples at the replicate level is 1·622 (95% CI 1·422–1·850).

Gametocyte quantification. qRT-PCR assay was used to measure gametocyte-specific *pvs25* mRNA transcripts (Genbank accession GU971513) (Table S3). *In silico* analysis for specificity showed 100% identity with gene targets, with no substantial predicted off-target interactions.

qRT-PCR was conducted with One-Step RT-PCR mix (Qiagen, Australia) using methods previously described[6,7] with 0·45 µM of each primer and 0·18 µM of Taqman probe in each qRT-PCR reaction. Amplification was performed in a Rotorgene 3000 or Q instrument (Qiagen, Australia) under the following cycling conditions: 50°C reverse transcription for 30 min, 95°C incubation for 15 min, followed by 45 cycles of 95°C for 15 sec and 60°C for 60 sec. Additional PCR reactions with heat inactivated reverse transcriptase were included to ensure genomic DNA had been eliminated. Quantitation was achieved using standard curves generated from serial-diluted synthetic RNA (synRNA) controls. SynRNA controls were manufactured into synthetic linear dsDNA with a T7 promoter attached (Thermo Fisher Scientific, Australia). In vitro transcribed RNA was made from the synthetic linear DNA using HiScribe T7 High Yield RNA Synthesis Kit (New England BioLabs) following manufacturer's instructions and subjected to two treatments with DNase digestion (RNase-free DNase set, Qiagen, Australia) to eliminate synthetic DNA contamination. SynRNA was purified using the Qiagen RNA mini kit (Qiagen, Australia), and neat synRNA quantitated using the High Sensitivity RNA Qubit assay (Thermo Fisher Scientific). Serial dilutions (*pvs25*: $2{\cdot}34\times10^6$ to 2·34 synRNA copies/µL) were prepared in uninfected human whole blood extracts and analysed in replicates to generate standard curves for transcript quantification.

P vivax gametocytes were measured by quantifying *pvs25* mRNA transcripts in whole blood. In the absence of pure *P vivax* gametocyte standards to quantify gametocyte numbers, we used the conversion factor of 35 *pvs25* mRNA transcripts per gametocyte previously determined by Karl et al.[8] to convert *pvs25* transcripts/mL to female gametocytes/mL.

Oligo name	Sequences	Target	Reference
**Saf 18S**	5'- AGGAAGTTTAAGGCAACAACAGGT -3'<br>5'- GCAATAATCTATCCCCATCACGA -3'<br>5'- FAM-TGTCCTTAGATGAACTAGGCTGCACGCG-BHQ1-3'	18S rRNA gene	[6]
pvs25	5'- ACCTTTCCGAAAATACATGTGAAGA-3'<br>5'- GGGTTTTCTATACACTGGCCAAAT-3'<br>5'- FAM-AGAAAGAAACCCTAGGCAAAGCATGCGG-BHQ1-3'	*pvs25* mRNA transcript	[6]

Table S3: Primers and probes used in the qRT-PCR assays

Estimating inoculum size for volunteer infection studies using *P vivax* (Bank HMP013) (includes figure S1)

Methods. The increase in parasite concentration measured between the day of infection (Day 0) and the day of treatment (Day 8, 9, or 10) was used to extrapolate an estimate of the parasite concentration in participants immediately after inoculation. Following the approaches of others,[2] we fit a log-linear model of parasite growth to the parasite concentration data collected from individuals. This assumes that parasites grow exponentially. When qPCR quantification of parasite concentration identified no parasite DNA in a sample this was termed a "non-detect" (ND). We identified that the treatment of ND had a significant impact on the estimates of the inoculum size. Accordingly, we adopted a censored regression approach where ND and other values detected below the limit of detection (LOD, 64 parasites/mL) were censored from the regression. This is a well-established method of dealing with limits of detection.[3]

The regression model was fit separately to each individual. Replicate qPCR measurements of parasite concentration, taken at the same time point, were all included in the analysis, and were not aggregated prior to regression. Raw replicate measurements were used because, at some time points, one replicate measurement of parasite concentration showed an ND, while other replicate measurements determined a parasite concentration above the LOD (figure S1). The software package, R (version 3.3.3) with package 'censReg'[4] was used to perform the censored regression analysis.

The estimated inoculum size for the two studies was taken as the mean of each individual's estimated inoculum size, and a t-distribution was used to determine the confidence interval of this mean across individuals.

Once an estimate of the initial parasite concentration was attained, $[P_0]$, we estimated the number of parasites injected into individuals (inoculum size), P_0, by assuming participants had an average blood volume of 5L. It follows that the inoculum size is given by:

$$P_0 = 5000 \times [P_0]$$

However, as a result of the multiple copies of the 18S RNA gene in the *P vivax* genome, this value must be adjusted by dividing by the scaling factor (detailed on page 13). Therefore, the adjusted estimate of the inoculum size is given by

$$P_0 = 5000 \times [P_0]/1.622.$$

Results. The parasite concentration measurements over time for all participants (N=26) in these VIS are shown in Figure S1. We estimate that the parasite concentration immediately after inoculation was 0·11 (95% CI: 0·18–0·30) parasites/mL. Assuming an average human has a blood volume of 5L and adjusting for double counting of parasites, we estimate that subjects received ~564 (95% CI: 342–930) viable parasites initially.

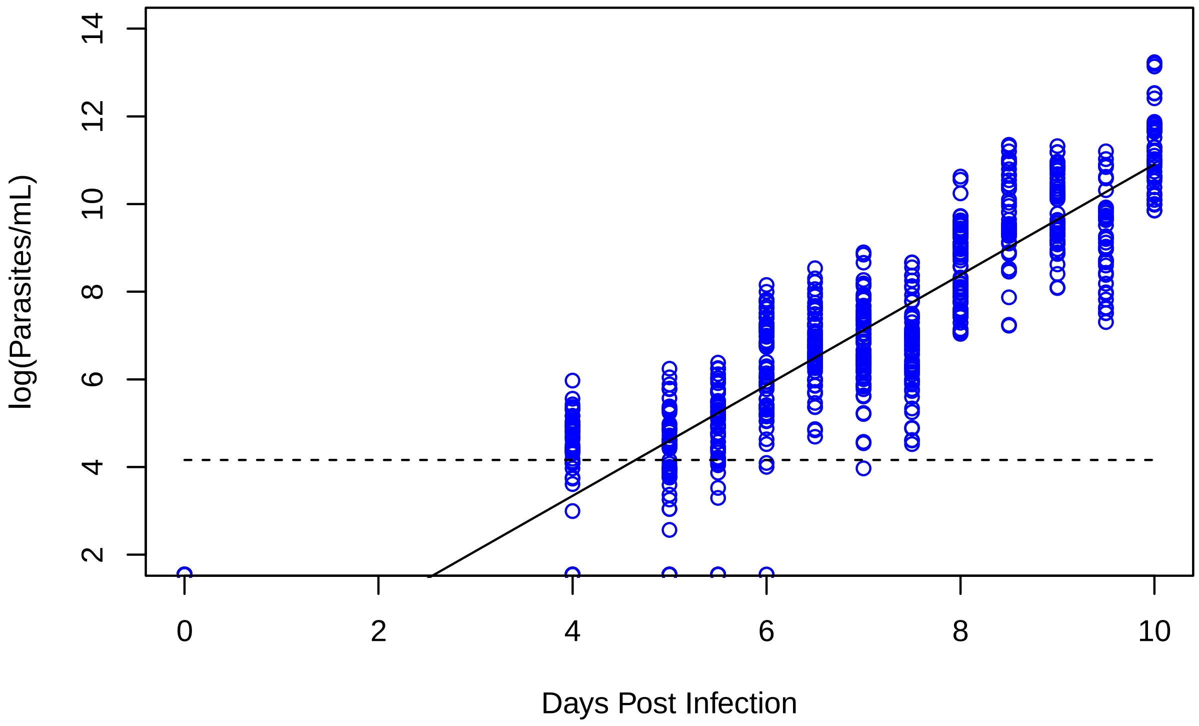

Figure S1. Parasite concentration (natural logarithm) from 26 participants over the course of the VIS (n=2 participants treated on Day 8 with artemether-lumefantrine, and N=24 participants treated with chloroquine [n=8 on Day 8, n=1 on Day 9, n=15 on Day 10]). The replicate measurements presented on the x-axis represent qPCR measurements that found no detectable parasites (ND). The dashed line indicates the limit of detection of the qPCR assay (64 parasites/mL). Parasite concentration can only reliably be measured above this limit. In the censored regression model, parasite concentrations below this limit (including NDs) were censored. The solid black line shows the average slope and intercept of all individuals, as determined by fitting the censored regression model.

Anopheles stephensi mosquito colony and feeding assays

Anopheles stephensi (Sind-Kasur Nijmegen strain)[9] mosquitoes were reared in the QIMR Berghofer insectary and mosquito feeding assays performed as described previously.[7] Briefly, mosquitoes were reared at 30°C, 70–80% relative humidity, exposed to a 12:12 h day:night light cycle. Mosquitoes 3–5 days post emergence were used for all feeding assays and were starved for ~12 h prior to feeding. Post feeding assay, the adult mosquitoes were maintained at 27°C, 70–80% relative humidity, until dissection. For direct feeding assays (DFA), ~ 30 mosquitoes were placed into small plastic containers with gauze lids and allowed to feed directly on the skin of participants for ~15 min. For all membrane feeding assays, 50–100 female mosquitoes were placed into pint containers with gauze lids. For direct membrane feeding assays (DMFA), venous blood was collected in lithium heparin vacutainers and mosquitoes were allowed to feed on whole blood via a glass membrane feeding device attached to a 37°C circulating water bath for ~30 min. For membrane feeding after replacement of participant's plasma with control AB serum (MFA-SR), whole blood was centrifuged for 1 min at 1000 g in a pre-warmed centrifuge and plasma removed. Control AB serum (from a malaria-naïve donor) was added and mixed with the red blood cells (RBCs) and the feed carried out as for DMFA.

For percoll enrichment experiments, whole blood samples pooled from multiple participants were leukodepleted and then layered onto a 65% percoll gradient and centrifuged for 15 min at 1500 g. The gametocyte containing band was removed from the gradient and washed 3 times in RPMI media. The gametocyte pellet was reconstituted in control O+ RBCs (Australian Red Cross Blood Service) and control AB serum (50% haematocrit) at a 10th or 40th of the starting blood volume.

For oocyst detection, ~50–100 mosquitoes per membrane feeding assay (DMFA or MFA SR) and ~30 mosquitoes per DFA were dissected 8 or 9 days post feeding assay. Mosquito midguts were removed and stored in 180 µL DNA Tissue Lysis buffer (Roche Diagnostics, Australia) either immediately or following microscopic analysis of the midgut stained with 0·5% mercurochrome. Next, 20 µL of Proteinase K (Qiagen, Australia) was added to the lysis mixture and incubated at 56°C overnight. Total nucleic acid extraction was performed the next day using the MagNA Pure 96 instrument (Roche Diagnostics, Australia) with Viral NA Small Volume Kit following the manufacturer's protocol (DNA Tissue S2.0)

The QuantiNova Probe PCR Kit (Qiagen, Australia) was used for all midgut testing for presence of malarial DNA using 18S rDNA qPCR assay described on page 15 of this supplementary appendix. Each 10µL PCR reaction mix consisted of 0·4 µM of each primer, 0.2µM of probe, QuantiNovaRox reference dye (1:200) and 4µL of template DNA. Amplification was performed on a ViiA7 Real-Time PCR System (Life Technologies, Australia) with the following cycling conditions: 95°C heat activation for 2 min, 45 fast cycles of 95°C for 5 sec and 60°C for 5 sec. Successful transmission was defined as at least one oocyst-positive mosquito per experimental feed and is reported as the percentage of the mosquitoes infected in each feeding experiment (prevalence of infection).[10]

For sporozoite quantification, mosquitoes were dissected and their salivary glands were removed and gently disrupted in a tissue homogeniser and counted on a haemocytometer.

Liver invasion assays

Cell line maintenance. HC-04 cells (BEI Resources, Manassas, VA) were maintained in complete culture media composed of 1:1 Modified Eagle's Medium (MEM) : F-12 Nutrient Mixture (Thermo Fisher Scientific Waltham, MA), supplemented with 10% FBS, 15 mM HEPES, 1·5 g/l sodium bicarbonate, 2·5 mM L-glutamine. Cultures were maintained in a humidified incubator at 37°C, 5% CO2. Cells were subcultured once a confluency of 70–90% was reached using Accutase® (Thermo Fisher Scientific, Waltham, MA) to disassociate the monolayer and passaged at a 1:3 ratio for a maximum of 15 times.

HC-04 Infection with *P vivax* sporozoites. Host HC-04 cells were harvested and resuspended in complete media and seeded at 10000 cells/well in 50 µl in a 384-well, TC-treated CellCarrier plates (PerkinElmer, Waltham, MA), 24 h prior to the inoculation with *P vivax* sporozoites.

Mosquitoes (15 days after blood feeding on enriched gametocytes collected on day 10 from participants in cohort 3) were dissected in Schneider's medium (Thermo Fisher Scientific, Waltham, MA) and whole salivary glands were collected in microfuge tubes and homogenised in ice. Viable parasites were counted using a haemocytometer. The sporozoite suspension was then pelleted at 4°C and resuspended in fresh Schneider's medium.

40 µl of media were removed from wells containing the host cells, and replaced with 25 µl of sporozoite suspension at a host cell:sporozoite ratio of 1:4, corresponding to 40000 *P vivax* sporozoites/well. Plates were centrifuged at 3200 rpm for 15 min at 4° C to accelerate sporozoite interaction with cell monolayer, then 70 µl of complete media was added to the wells. The plates were incubated for 4 h at 37° C, 5% CO2 to allow the sporozoites to invade the host cells. The media and remaining extracellular sporozoites were then removed and replaced with 50 µl of complete media containing Penicillin-Streptomycin (200 U/ml) (Thermo Fisher Scientific, Waltham, MA). Following 24 h incubation, the media was replenished with Penicillin-Streptomycin media (100 U/ml) and then routine media changes were performed every 48 h until assay endpoints were reached.

Immunofluorescence microscopy assessment of *P. vivax* infected HC-04 cells. After 7 days incubation, plates were fixed with 4% paraformaldehyde (PFA) for 20 min, washed three times with PBS, and blocked/permeabilised overnight at 4 °C with 2% bovine serum albumin (BSA) and 0.2% Triton-x (Sigma-Aldrich, St. Louis, MO). Primary UIS4 monoclonal mouse antibody that localises to the parasitophorous vacuole membrane of infected cells (Center for Infectious Disease Research, Seattle, WA) was diluted 1/400 in blocking buffer (2% BSA and 0.2% Triton-x in PBS) and then added to the cells and incubated overnight at 4°C. The next day the plates were washed three times with PBS and 50 µl of a mixture of secondary alexa fluor 488-conjugated anti-mouse antibody (Thermo Fisher Scientific, Waltham, MA) (1/400) and Hoechst 33342 (20 µM) in blocking buffer was added and incubated for 2 h at room temperature with gentle shaking. At the end of the incubation, the unbound secondary antibody was removed with 3× PBS washes and kept in 50 µl of PBS for imaging and long-term storage.

The plates were imaged using an Opera Confocal High-Content Imaging system (PerkinElmer, Waltham, MA), with a 20× water immersion objective and 405 nm excitation and 450/40 nm emission for Hoechst and 488 nm excitation and 540/35 emission for UIS4. A 7-planes Z-stack at a resolution of 5 µm, with 63 fields covering the entire well, was acquired. Image analysis was performed with the PerkinElmer Acapella® based Opera or Columbus software using standard supervised feature selection and segmentation of nuclei and the positive UIS4 immunofluorescent staining. Positive identification of infected HC-04 cells was determined by the selection of objects that positively expressed UIS4 levels above background intensity levels given by secondary antibody only, and that contained multiple nuclei within the region of interest. Higher resolution images of positively identified liver-stage schizonts were obtained using a 40× water immersion objective with a 36-planes Z-stack at a resolution of 0.6 µm, using the same emission/excitation filter sets. To prepare Figure 4 the images were cropped and brightness/contrast adjusted using Adobe Photoshop CS6 (Adobe Systems, San Jose, CA).

Recalibration of the *P vivax* standard curve used in previous QIMR Berghofer *P vivax* IBSM studies (includes Figure S2)

Due to technical improvements in methods for production and quantification synthetic RNA, *pvs25* standard curves were re-derived for use in *pvs25* qRT-PCR assays at QIMR Berghofer. To enable re-calibration of the data generated using the old standard curve, quantification of samples using both standards was compared and a conversion factor determined.

The new standards were generated by producing synthetic linear dsDNA containing the qRT-PCR target sequence with T7 promoter (Thermo Fisher Scientific, Australia) and in vitro transcribing them to cRNA using HiScribe™ T7 High Yield RNA Synthesis Kit (New England BioLabs) following manufacturer's instructions. The product was subjected to two treatments with DNase digestion (RNase-free DNase set, Qiagen, Australia) to eliminate synthetic DNA contamination. cRNA was then purified using the Qiagen RNeasy mini kit (Qiagen, Australia). Quantity (numbers of transcripts) of neat cRNA control was calculated based on molecular weight measured by the High Sensitivity RNA Qubit assay (Thermo Fisher Scientific). Standard curves were generated by serially diluting the synthetic cRNA in uninfected human whole blood extracts to reflect similar matrix specimen type from clinical trials. To compare quantification using the new standard curve to the old standard curve, 9 *pvs25* positive samples (mRNA extracted from whole blood) at a range of gametocyte densities were analysed in duplicate on 3 separate days and the copy numbers per sample quantified using both standard curves. Linear regression was then used to compare quantification using both assays. The slope was equal to 1 (1·007, CI 95%: 0·9885–1·025) and thus the relationship between the two measures was constant. The slope was then constrained to 1 and non-linear regression was performed. The y-intercept was 2·508 ($\log_{10}$), which is an anti-log of 322 (figure S2). *Pvs25* transcript numbers determined by qRT-PCR during previous clinical studies at QIMR Berghofer [6,11] have therefore been amended by dividing the reported number by 322 to convert them to quantification using the new more accurate synthetic RNA standard curve.

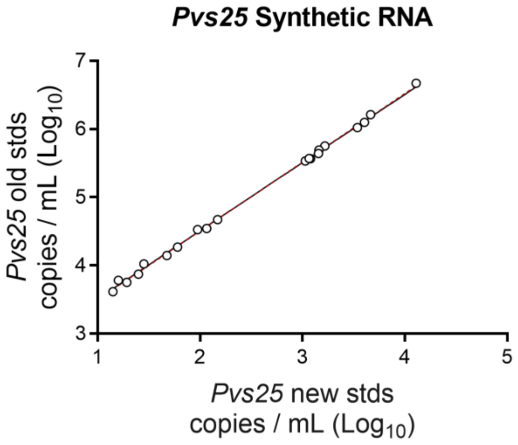

Figure S2. Comparison of quantification using the old and new synthetic RNA standard curve. Solid black line shows the linear regression. The dashed red line shows the non-linear regression.

SUPPLEMENTARY RESULTS

Cohort	Participant	Date of inoculation	Dose days and chloroquine doses (mg/kg)							
			Day of CQ dose 1	CQ Dose 1	Day of CQ dose 2	CQ Dose 2	Day of CQ dose 3	CQ Dose 3	Day of CQ dose 4	CQ Dose 4
**1**	101	02-MAR-2016	Day 8 am	10	Day 8 pm	5	Day 9 am	5	Day 10 am	5
	102	02-MAR-2016	Day 8 am	10	Day 8 pm	5	Day 9 am	5	Day 10 am	5
	103	02-MAR-2016	Day 8 am	10	Day 8 pm	5	Day 9 am	5	Day 10 am	5
	104	02-MAR-2016	Day 8 am	10	Day 8 pm	5	Day 9 am	5	Day 10 am	5
	105	02-MAR-2016	Day 8 am	10	Day 8 pm	5	Day 9 am	5	Day 10 am	5
	106	02-MAR-2016	Day 8 am	10	Day 8 pm	5	Day 9 am	5	Day 10 am	5
	107	02-MAR-2016	Day 8 am	10	Day 8 pm	5	Day 9 am	5	Day 10 am	3·75
	108	02-MAR-2016	Day 8 am	10	Day 8 pm	5	Day 9 am	5	Day 10 am	5
**2a**	201	06-FEB-2017	Day 10 am	10	Day 10 pm	5	Day 11 am	5	Day 12 am	5
	202	06-FEB-2017	Day 10 am	10	Day 10 pm	5	Day 11 am	5	Day 12 am	5
	203	06-FEB-2017	Day 10 am	10	Day 10 pm	5	Day 11 am	5	Day 12 am	5
	204	06-FEB-2017	Day 10 am	10	Day 10 pm	5	Day 11 am	3·75	Day 12 am	3·75
	205	06-FEB-2017	Day 9 am	10	Day 9 pm	5	Day 10 am	5	Day 11am	5
	206	06-FEB-2017	Day 10 am	10	Day 10 pm	5	Day 11 am	5	Day 12 am	5
**2b**	207	27-FEB-2017	Day 10 am	10	Day 10 pm	5	Day 11 am	5	Day 12 am	5
	208	27-FEB-2017	Day 10 am	10	Day 10 pm	5	Day 11 am	5	Day 12 am	5
**3**	301	27-MAR-2017	Day 10 am	10	Day 10 pm	5	Day 11 am	5	Day 12 am	5
	302	27-MAR-2017	Day 10 am	10	Day 10 pm	5	Day 11 am	5	Day 12 am	5
	303	27-MAR-2017	Day 10 am	10	Day 10 pm	5	Day 11 am	5	Day 12 am	5
	304	27-MAR-2017	Day 10 am	10	Day 10 pm	5	Day 11 am	5	Day 12 am	5
	305	27-MAR-2017	Day 10 am	10	Day 10 pm	5	Day 11 am	5	Day 12 am	5
	306	27-MAR-2017	Day 10 am	10	Day 10 pm	5	Day 11 am	5	Day 12 am	5
	307	27-MAR-2017	Day 10 am	10	Day 10 pm	5	Day 11 am	5	Day 12 am	5
	308	27-MAR-2017	Day 10 am	10	Day 10 pm	5	Day 11 am	5	Day 12 am	5

Table S4: Participant inoculation and chloroquine treatment (Study 2)

All participants in Study 2 were inoculated with ~564 *P vivax*-infected RBCs followed by treatment with chloroquine as detailed.

System Organ Class<br>Preferred term	Study 1	Study 2			Total
	n=2<br>n (%) M	Cohort 1<br>n=8<br>n (%) M	Cohort 2<br>n=8<br>n (%) M	Cohort 3<br>n=8<br>n (%) M	n=26<br>n (%) M
**Blood and lymphatic system disorders**					
Splenomegaly	0	0	1 (12·5%) 1	1 (12·5%) 1	2 (7·7%) 2
**Cardiac disorders**					
Presyncope	1 (50·0%) 1	0	0	0	1 (3·8%) 1
Tachycardia	0	0	1 (12·5%) 1	1 (12·5%) 1	2 (7·7%) 2
**Ear and labyrinth disorders**					
Ear pain	0	0	0	1 (12·5%) 1	1 (3·8%) 1
**Eye disorders**					
Conjunctival hyperemia	0	0	0	1 (12·5%) 1	1 (3·8%) 1
**Gastrointestinal disorders**					
Abdominal discomfort	0	1 (12·5%) 1	4 (50·0%) 6	4 (50·0%) 4	9 (34·6%) 11
Abdominal tenderness	0	0	0	1 (12 ·5%) 1	1 (3·8%) 1
Diarrhoea	0	0	1 (12·5%) 2	0	1 (3·8%) 1
Nausea	0	3 (37·5%) 6	3 (37·5%) 8	7 (87·5%) 12	13 (50·0%) 26
Vomiting	0	1 (12·5%) 1	3 (37·5%) 5	2 (25·0%) 2	6 (23·1%) 8
**General disorders and administration site conditions**					
Chills	0	1 (12·5%) 1	4 (50·0%) 8	4 (50·0%) 8	9 (34·6%) 17
Fatigue	0	3 (37·5%) 5	4 (50·0%) 7	7 (87·5%) 15	14 (53·8%) 27
Influenza-like illness	0	1 (12·5%) 2	4 (50·0%) 7	2 (25·0%) 4	7 (26·9%) 13
Malaise	0	0	4 (50·0%) 6	7 (87·5%) 9	11 (42·3%) 15
Pyrexia/fever	2 (100%) 2	3 (37·5%) 4	7 (87·5%) 10	7 (87 ·5%) 15	19 (73·1%) 31
Rigors	1 (50·0%) 1	0	0	0	1 (3·8%) 1
Somnolence	1 (50·0%) 1	0	0	0	1 (3·8%) 1
Vessel puncture site bruise	0	1 (12·5%) 2	3 (37·5%) 3	4 (50.0%) 7	8 (30·8%) 12
Vessel puncture site pain	0	0	0	1 (12.5%) 1	1 (3·8%) 1
**Infections and infestations**					
Pyelonephritis	0	0	0	1 (12·5%) 1	1 (3·8%) 1
Tooth abscess	0	0	0	1 (12·5%) 1	1 (3·8%) 1
Upper respiratory tract infection	0	2 (25·0%) 2	0	1 (12·5%) 2	3 (11·5%) 4
**Injury, poisoning and procedural complications**					
Arthropod bite	0	1 (12·5%) 1	5 (62·5%) 7	3 (37·5%) 3	9 (34·6%) 11
**Investigations**					
Alanine aminotransferase increased	0	0	2 (25·0%) 2	2 (25·0%) 3	4 (15·4%) 5
Aspartate aminotransferase increased	0	0	0	1 (12·5%) 1	1 (3·8%) 1
Lymphocyte count decreased	0	0	6 (75·0%) 6	5 (62·5%) 5	11 (42·3%) 11
Neutrophil count decreased	0	0	3 (37·5%) 4	2 (25·0%) 2	5 (19·2%) 6
Platelet count decreased	0	0	1 (12·5%) 1	0	1 (3·8%) 1
White blood cell count decreased	0	0	3 (37·5%) 3	2 (25·0%) 2	5 (19·2%) 5
**Metabolism and nutrition disorders**					
Decreased appetite	0	2 (25·0%) 2	3 (37·5%) 7	5 (62·5%) 7	10 (38·5%) 16
Dehydration	0	0	2 (25·0%) 2	0	2 (7·7%) 2
Hypocalcaemia	0	0	1 (12·5%) 1	0	1 (3·8%) 1
Hypoglycaemia	0	0	1 (12·5%) 1	0	1 (3·8%) 1

System Organ Class<br>Preferred term	Study 1	Study 2			Total<br>n=26<br>n (%) M
	n=2<br>n (%) M	Cohort 1<br>n=8<br>n (%) M	Cohort 2<br>n=8<br>n (%) M	Cohort 3<br>n=8<br>n (%) M	
**Musculoskeletal and connective tissue disorders**					
Arthralgia	1 (50·0%) 1	1 (12·5%) 1	4 (50·0%) 11	4 (50·0%) 7	9 (34·6%) 19
Myalgia	1 (50·0%) 2	4 (50·0%) 6	6 (75·0%) 11	7 (87·5%) 10	18 (69·2%) 29
**Nervous system disorders**					
Dizziness	0	0	2 (25·0%) 2	1 (12·5%) 1	3 (11·5%) 3
Headache	2 (100%) 6	6 (75·0%) 10	8 (100%) 33	7 (87·5%) 16	23 (88·5%) 65
Lethargy	0	0	0	1 (12·5%) 2	1 (3·8%) 2
**Psychiatric disorders**					
Insomnia	0	0	0	1 (12·5%) 1	1 (3·8%) 1
**Reproductive system and breast disorders**					
Vulvovaginal pruritus	0	0	0	1 (12·5%) 1	1 (3·8%) 1
**Respiratory, thoracic and mediastinal disorders**					
Oropharyngeal pain	0	0	0	1 (12·5%) 2	1 (3·8%) 2
Rhinorrhoea	0	0	0	3 (37·5%) 3	3 (11·5%) 3
**Skin and subcutaneous tissue disorders**					
Pruritus	0	1 (12·5%) 1	0	0	1 (3·8%) 1

Table S5: All adverse events by system organ class and preferred term

If a participant had multiple occurrences of an AE, the participant is presented only once in the participant count (n) column for a given System Organ Class and Preferred Term. Occurrences are counted each time in the mentions/occurrence (M) column.

Cohort 1		Mosquito infection rate<br>No. mosquitoes infected/no. mosquitoes tested (%)							
**Day**	**Assay \ Participant**	**101**	**102**	**103**	**104**	**105**	**106**	**107**	**108**
**6**	**DFA**	NP	NP	NP	NP	NP	NP	NP	NP
	**DMFA**	0/50 (0)	0/50 (0)	0/48 (0)	0/50 (0)	0/50 (0)	0/50 (0)	0/50 (0)	0/50 (0)
	**MFA-SR**	NP	NP	NP	NP	NP	NP	NP	NP
**7**	**DFA**	0/30 (0)	0/30 (0)	0/30 (0)	0/30 (0)	0/30 (0)	0/30 (0)	0/30 (0)	0/30 (0)
	**DMFA**	0/50 (0)	0/48 (0)	0/46 (0)	0/50 (0)	0/50 (0)	0/43 (0)	0/49 (0)	0/50 (0)
	**MFA-SR**	NP	NP	NP	NP	NP	NP	NP	NP
**8**	**DFA**	0/30 (0)	0/30 (0)	0/30 (0)	0/26 (0)	0/30 (0)	0/30 (0)	0/30 (0)	0/30 (0)
	**DMFA**	0/50 (0)	0/50 (0)	0/50 (0)	0/50 (0)	0/50 (0)	0/50 (0)	**1/50 (2.0)**	0/50 (0)
	**MFA-SR**	NP	NP	NP	NP	NP	NP	NP	NP
**Cohort 2**		**Mosquito infection rate<br>No. mosquitoes infected/no. mosquitoes tested (%)**							
**Day**	**Assay \ Participant**	**201**	**202**	**203**	**204**	**205**	**206**	**207**	**208**
**9**	**DFA**	**1/34 (2·9)**	0/30 (0)	0/33 (0)	**1/34 (2·9)**	0/33 (0)	0/31 (0)	0/36 (0)	**1/29 (3·4)**
	**DMFA**	0/118 (0)	**1/72 (1·4)**	0/76 (0)	**2/72 (2·8)**	0/78 (0)	0/90 (0)	0/104 (0)	0/101 (0)
	**MFA-SR**	0/89 (0)	**1/82 (1·2)**	0/70 (0)	**9/104 (8·6)**	NP	NP	0/99 (0)	0/113 (0)
**10**	**DFA**	0/35 (0)	**2/34 (5·9)**	0/31 (0)	0/35 (0)	NP	0/32 (0)	0/31 (0)	0/34 (0)
	**DMFA**	0/117 (0)	**1/107 (0·9)**	0/109 (0)	**7/112 (6·3)**	NP	0/78 (0)	0/98 (0)	0/91 (0)
	**MFA-SR**	0/111 (0)	**5/110 (4·5)**	0/110 (0)	**20/110 (18·2)**	NP	0/90 (0)	**8/82 (9·7)**	0/121 (0)
**Cohort 3**		**Mosquito infection rate<br>No. mosquitoes infected/no. mosquitoes tested (%)**							
**Day**	**Assay \ Participant**	**301**	**302**	**303**	**304**	**305**	**306**	**307**	**308**
**9**	**DFA**	0/29 (0)	0/30 (0)	**1/31 (3·2)**	**1/34 (2·9)**	0/30 (0)	0/34 (0)	NP	0/20 (0)
	**DMFA**	0/70 (0)	0/101 (0)	0/50 (0)	**1/62 (1·6)**	0/56 (0)	0/58 (0)	NP	0/81 (0)
	**MFA-SR**	NP	NP	NP	NP	NP	NP	NP	NP
**10**	**DFA**	**4/31 (12·9)**	**2/30 (6·7)**	**1/29 (3·4)**	0/30 (0)	0/33 (0)	0/33 (0)	**1/35 (2·8)**	0/34 (0)
	**DMFA**	0/71 (0)	**3/107 (2·8)**	0/96 (0)	0/87 (0)	0/103 (0)	0/105 (0)	0/111 (0)	**1/94 (1·1)**
	**MFA-SR**	NP	NP	NP	NP	NP	NP	NP	NP

Table S6: Participant infectivity to mosquitoes (Study 2)

Infectivity to mosquitoes of each participant during feeding assays in Study 2. Infection is defined as presence of oocysts in the mosquito midgut on Day 8 or 9 after feeding assay as determined by 18S rDNA qPCR. Mosquito infection rate is reported as prevalence of infection (percentage of mosquitoes infected per assay). Assays with infected mosquitoes in bold.

DFA=direct feeding assay; DMFA=direct membrane feeding assay; MFA-SR=membrane feeding assay with serum replacement; NP=not performed.

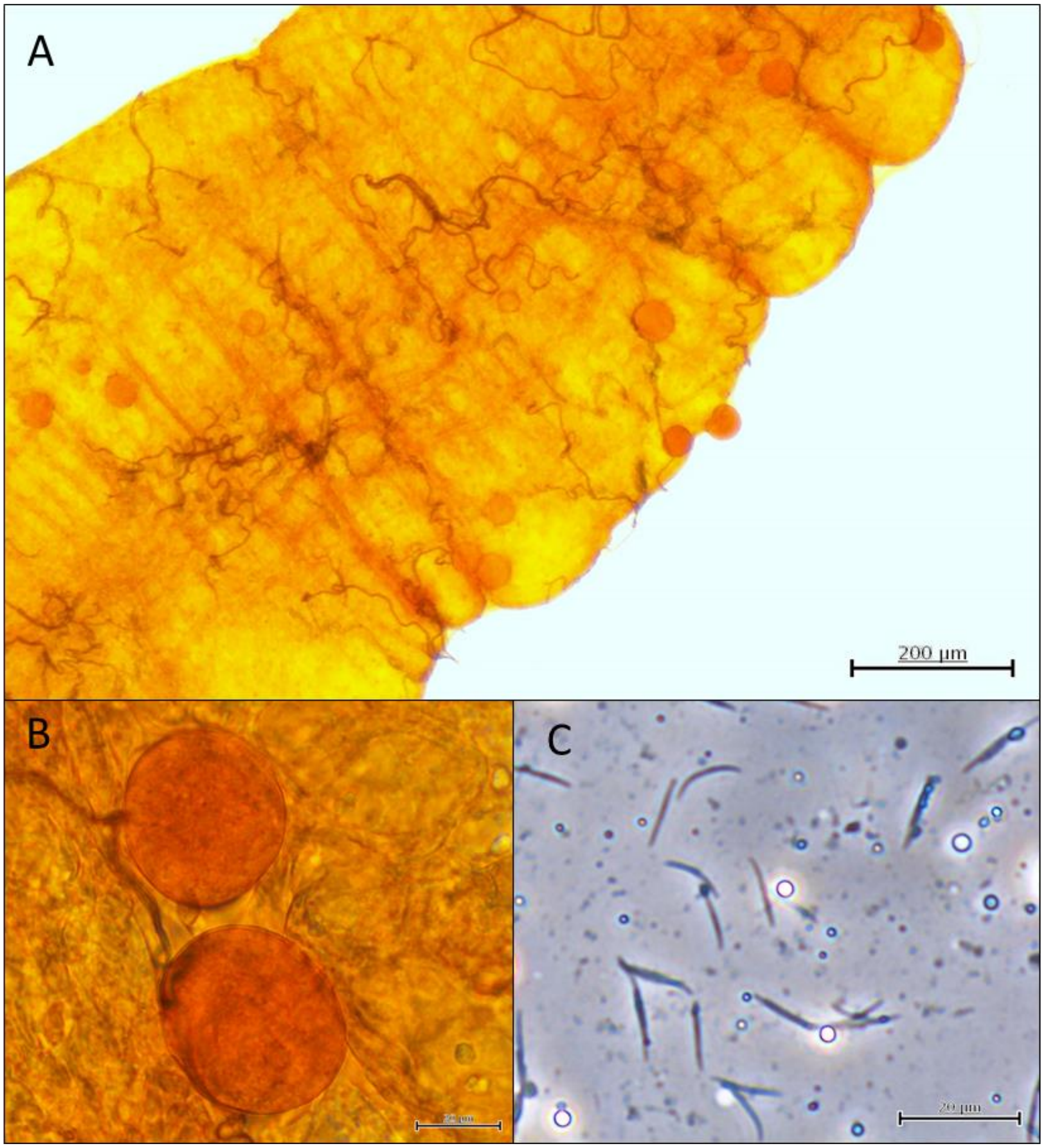

Figure S3. Oocysts and sporozoites visualised by microscopy

(A+B) Midguts dissected and stained with 0·5% mercurochrome to visualise the oocysts from mosquitoes fed on percoll-enriched gametocytes. (C) Sporozoites visualised by phase contrast microscopy from a mosquito fed on percoll-enriched gametocytes.

References

1. Gaziano TA, Young CR, Fitzmaurice G, Atwood S, Gaziano JM. Laboratory-based versus non-laboratory-based method for assessment of cardiovascular disease risk: the NHANES I follow-up study cohort. *Lancet* 2008; **371**(9616): 923-31.
2. Douglas AD, Edwards NJ, Duncan CJ, et al. Comparison of modeling methods to determine liver-to-blood inocula and parasite multiplication rates during controlled human malaria infection. *J Infect Dis* 2013; **208**(2): 340-5.
3. Tobin J. Estimation of relationships for limited dependents variables. *Econometrica* 1958; **26**(1): 26-36.
4. Henningsen A. censReg: Censored regression (Tobit) models. R package version 0.5. http://CRAN.R-Project.org/package=censReg.
5. Rockett RJ, Tozer SJ, Peatey C, et al. A real-time, quantitative PCR method using hydrolysis probes for the monitoring of *Plasmodium falciparum* load in experimentally infected human volunteers. *Malar J* 2011; **10**(1): 1-6.
6. McCarthy JS, Griffin PM, Sekuloski S, et al. Experimentally induced blood-stage *Plasmodium vivax* infection in healthy volunteers. *J Infect Dis* 2013; **208**(10): 1688-94.
7. Collins KA, Wang CY, Adams M, et al. A controlled human malaria infection model enabling evaluation of transmission-blocking interventions. *J Clin Invest* 2018; **128**(4): 1551-62.
8. Karl S, Laman M, Koleala T, et al. Comparison of three methods for detection of gametocytes in Melanesian children treated for uncomplicated malaria. *Malar J* 2014; **13**: 319.
9. Feldmann AM, Ponnudurai T. Selection of *Anopheles stephensi* for refractoriness and susceptibility to Plasmodium falciparum. *Med Vet Entomol* 1989; **3**(1): 41-52.
10. Wang CYT, McCarthy JS, Stone WJ, Bousema T, Collins KA, Bialasiewicz S. Assessing *Plasmodium falciparum* transmission in mosquito-feeding assays using quantitative PCR. *Malar J* 2018; **17**(1): 249.
11. Griffin P, Pasay C, Elliott S, et al. Safety and reproducibility of a clinical trial system using induced blood stage *Plasmodium vivax* infection and its potential as a model to evaluate malaria transmission. *PLoS Negl Trop Dis* 2016; **10**(12): e0005139.

CONSORT 2010 checklist of information to include when reporting a randomised trial*

Section/Topic	Item No	Checklist item	Reported on page No
**Title and abstract**			
	1a	Identification as a randomised trial in the title	N/A
	1b	Structured summary of trial design, methods, results, and conclusions (for specific guidance see CONSORT for abstracts)	2
**Introduction**			
Background and objectives	2a	Scientific background and explanation of rationale	4-5
	2b	Specific objectives or hypotheses	5
**Methods**			
Trial design	3a	Description of trial design (such as parallel, factorial) including allocation ratio	12-14
	3b	Important changes to methods after trial commencement (such as eligibility criteria), with reasons	12-14
Participants	4a	Eligibility criteria for participants	14 + S3-8
	4b	Settings and locations where the data were collected	12
Interventions	5	The interventions for each group with sufficient details to allow replication, including how and when they were actually administered	N/A
Outcomes	6a	Completely defined pre-specified primary and secondary outcome measures, including how and when they were assessed	13-14
	6b	Any changes to trial outcomes after the trial commenced, with reasons	N/A
Sample size	7a	How sample size was determined	14
	7b	When applicable, explanation of any interim analyses and stopping guidelines	N/A
Randomisation:			
Sequence generation	8a	Method used to generate the random allocation sequence	N/A
	8b	Type of randomisation; details of any restriction (such as blocking and block size)	N/A
Allocation concealment mechanism	9	Mechanism used to implement the random allocation sequence (such as sequentially numbered containers), describing any steps taken to conceal the sequence until interventions were assigned	N/A
Implementation	10	Who generated the random allocation sequence, who enrolled participants, and who assigned participants to interventions	N/A
Blinding	11a	If done, who was blinded after assignment to interventions (for example, participants, care providers, those	N/A

		assessing outcomes) and how	
	11b	If relevant, description of the similarity of interventions	N/A
Statistical methods	12a	Statistical methods used to compare groups for primary and secondary outcomes	14
	12b	Methods for additional analyses, such as subgroup analyses and adjusted analyses	N/A
**Results**			
Participant flow (a diagram is strongly recommended)	13a	For each group, the numbers of participants who were randomly assigned, received intended treatment, and were analysed for the primary outcome	6
	13b	For each group, losses and exclusions after randomisation, together with reasons	6
Recruitment	14a	Dates defining the periods of recruitment and follow-up	6
	14b	Why the trial ended or was stopped	N/A
Baseline data	15	A table showing baseline demographic and clinical characteristics for each group	23
Numbers analysed	16	For each group, number of participants (denominator) included in each analysis and whether the analysis was by original assigned groups	6-8
Outcomes and estimation	17a	For each primary and secondary outcome, results for each group, and the estimated effect size and its precision (such as 95% confidence interval)	6-8 and S20-24
	17b	For binary outcomes, presentation of both absolute and relative effect sizes is recommended	N/A
Ancillary analyses	18	Results of any other analyses performed, including subgroup analyses and adjusted analyses, distinguishing pre-specified from exploratory	N/A
Harms	19	All important harms or unintended effects in each group (for specific guidance see CONSORT for harms)	N/A
**Discussion**			
Limitations	20	Trial limitations, addressing sources of potential bias, imprecision, and, if relevant, multiplicity of analyses	11
Generalisability	21	Generalisability (external validity, applicability) of the trial findings	9-11
Interpretation	22	Interpretation consistent with results, balancing benefits and harms, and considering other relevant evidence	9-11
**Other information**			
Registration	23	Registration number and name of trial registry	2 + 14
Protocol	24	Where the full trial protocol can be accessed, if available	Supplementary
Funding	25	Sources of funding and other support (such as supply of drugs), role of funders	2 + 15

*We strongly recommend reading this statement in conjunction with the CONSORT 2010 Explanation and Elaboration for important clarifications on all the items. If relevant, we also recommend reading CONSORT extensions for cluster randomised trials, non-inferiority and equivalence trials, non-pharmacological treatments, herbal interventions, and pragmatic trials. Additional extensions are forthcoming: for those and for up to date references relevant to this checklist, see www.consort-statement.org.
